# Cell Type-Specific Downregulation of Dnmt3a in Nucleus Accumbens Oligodendrocytes Prevents Myelin Damage and Reduces Susceptibility to Social Stress in Male Mice

**DOI:** 10.3390/biom16050639

**Published:** 2026-04-24

**Authors:** Yifan Niu, Kaiwei Li, Kaiyuan Zhan, Mingshan Pi, Qi Xiong, Ji Wang, Xiaochuan Wang, Xiji Shu, Yiyuan Xia, Mengbing Huang

**Affiliations:** 1 Hubei Key Laboratory of Cognitive and Affective Disorders, Institutes of Biomedical Sciences, School of Medicine, Jianghan University, Wuhan 430056, China; nyfjhusy@163.com (Y.N.); likaiwei0114@163.com (K.L.); aisure7@163.com (K.Z.); pims.whibs@aliyun.com (M.P.); xiongq.whibs@aliyun.com (Q.X.); wangji6868@163.com (J.W.); wxch@mails.tjmu.edu.cn (X.W.); xijishu@jhun.edu.cn (X.S.); 2 Hubei Provincial Demonstration Center for Experimental Medicine Education, School of Medicine, Jianghan University, Wuhan 430056, China; 3 Department of Physiology, School of Medicine, Jianghan University, Wuhan 430056, China

**Keywords:** nucleus accumbens, oligodendrocytes, Dnmt3a, myelination, susceptibility to social stress

## Abstract

Background: Chronic stress is a major contributing factor to mood disorders, including depression and anxiety; however, the molecular mechanisms underlying individual differences in susceptibility to such disorders remain poorly understood. DNA methyltransferase 3a (Dnmt3a), a key epigenetic regulator, has been increasingly implicated in stress-related neurobiological adaptations. In this study, we employed a well-established mouse model of chronic social defeat stress (CSDS) to investigate the functional role of Dnmt3a in modulating individual susceptibility to social stress. Methods: Male C57BL/6J mice were exposed to chronic/submaximal social defeat stress (CSDS/SSDS). AAV vectors were used to achieve Dnmt3a overexpression or global and oligodendrocyte-specific knockdown in the nucleus accumbens (NAc). Behavioral tests, including social interaction, open field, and elevated zero maze, were conducted alongside Western blotting and immunofluorescence assays. Results: CSDS selectively increased Dnmt3a expression in NAc oligodendrocytes of stress-susceptible mice. Overexpression of Dnmt3a in the NAc enhanced susceptibility to stress, whereas its knockdown conferred resilience, without affecting baseline behaviors. Dnmt3a negatively regulated myelin basic protein (MBP) and dopamine D1 receptor expression. Stress-susceptible mice exhibited shortened myelinated segments and reduced D1 receptor levels, while D2 receptor expression remained unchanged. Conclusions: Dnmt3a in NAc oligodendrocytes modulates susceptibility to social stress through a Dnmt3a-MBP/D1 receptor-NAc pathway, highlighting a critical glia-neuron interaction. This mechanism extends our understanding of the neurobiological basis of stress-related disorders and positions Dnmt3a as a promising therapeutic target for developing precision interventions or biomarkers.

## 1. Introduction

Social stress refers to negative stimuli such as social rejection, interpersonal conflict, and defeat that individuals experience during social interaction (SI). Prolonged exposure to such stress can trigger a range of psychiatric disorders, with social anxiety disorder, major depressive disorder, and post-traumatic stress disorder being the most prevalent, collectively imposing a substantial burden on public mental health [1,2]. Although evidence supports the involvement of genetic factors, environmental influences, and their interplay in disease etiology, the precise underlying pathogenic mechanisms remain incompletely understood [3,4,5]. Current standard treatments, including selective serotonin reuptake inhibitors and cognitive behavioral therapy, are limited by suboptimal response rates, delayed therapeutic onset, and significant side effects [6]. Notably, 30% to 50% of patients fail to respond adequately to conventional therapies, highlighting the biological heterogeneity of these disorders and underscoring the urgent need to identify novel regulatory mechanisms and therapeutic targets [7]. Therefore, elucidating the molecular and cellular basis of susceptibility to social stress holds significant clinical promise for advancing the development of more effective diagnostic biomarkers and precision treatment strategies.

Individuals exhibit marked differences in susceptibility when exposed to the same intensity of social stress: some develop pathological phenotypes such as social avoidance and anxiety, classified as stress-susceptible, while others maintain normal social functioning and emotional homeostasis, defined as stress-resilient [8,9]. This individual variability is primarily attributed to functional plasticity within the brain’s stress-regulatory circuitry, in which the nucleus accumbens (NAc) and medial prefrontal cortex (mPFC) serve as central nodes [10]. The NAc, acting as a critical interface between the limbic and motor systems, integrates reward processing, emotional regulation, and social behavior, and its dysfunction is strongly associated with core symptoms of depression and anxiety disorders, including social withdrawal and anhedonia [10,11]. In contrast, the mPFC contributes to stress adaptation by modulating higher-order cognitive processes and emotional responses, thereby influencing the selection of behavioral coping strategies [12]. A growing body of evidence indicates that alterations in neuronal activity, neurotransmitter dynamics, and synaptic plasticity within the NAc and mPFC are critically involved in determining stress susceptibility [13,14,15,16]. Nevertheless, the precise molecular mechanisms underlying this regulation, particularly those operating in a brain region- and cell type-specific manner, remain poorly understood and require systematic investigation.

Epigenetic modifications are central mechanisms that mediate the interplay between environmental influences and gene expression. Among these, DNA methylation suppresses gene transcription by adding methyl groups to gene promoter regions and plays a critical role in the pathogenesis of stress-related psychiatric disorders [3,17,18]. DNA methyltransferases (Dnmts), including DNMT1, DNMT2, DNMT3a, DNMT3b, and DNMT3l, are the key enzymes responsible for catalyzing DNA methylation. However, only DNMT1, DNMT3a, and DNMT3b are capable of establishing global cytosine methylation in the human genome: DNMT1 maintains pre-existing methylation patterns during DNA replication, whereas DNMT3a and DNMT3b mediate de novo methylation, particularly during developmental and adaptive processes [19,20,21]. Accumulating evidence indicates that Dnmts are essential for brain development and neural function regulation, and their dysregulation is implicated in various neuropsychiatric conditions, including anxiety, depression, and schizophrenia [22,23]. For instance, overexpression of Dnmt3a in the mPFC reduces anxiety-like behaviors, while its knockdown exacerbates them; furthermore, elevated Dnmt3a expression in the mPFC of mice exposed to chronic stress mitigates stress-induced anxiety [24]; Similarly, forebrain-specific deletion of Dnmt1 confers resilience against both anxiety- and depression-like phenotypes [25]. Despite these advances, the role of Dnmts in regulating susceptibility to social stress, particularly their region- and cell type-specific functions in circuits such as the mPFC and NAc, remains poorly understood and has not been systematically investigated.

In this study, we investigated the expression patterns of Dnmts in the mPFC and NAc of mice subjected to chronic social stress, and examined their roles in regulating susceptibility to social stress and the underlying molecular mechanisms. We analyzed the differential expression of Dnmt1, Dnmt3a, and Dnmt3b in the mPFC and NAc. To further elucidate the functional significance of Dnmt3a, we employed AAV-mediated approaches to selectively knockdown or overexpress Dnmt3a in the NAc and assessed its effects on SI and anxiety-like behaviors. Additionally, we specifically knockdown Dnmt3a in oligodendrocytes within the NAc and evaluated the expression of myelin basic protein (MBP) and components of dopamine receptor signaling pathways.

## 2. Materials and Methods

### 2.1. Animals

Male C57BL/6J mice (6–7 weeks old) and male CD1 retired breeder mice (6–8 months old) were purchased from Beijing Vital River Laboratory Animal Technology Co., Ltd. (Beijing, China). The C57 mice were group-housed in groups of five, while the CD1 mice were individually housed. All animals were maintained under a 12 h light/dark cycle (8:30–20:30), with a constant temperature of 21 ± 2 °C, and provided ad libitum access to food and water except during behavioral tests. A one-week acclimation period was provided before experiments initiated at the Medical Animal Center of Jianghan University. All experimental procedures strictly adhered to the guidelines set forth by the Medical Ethics Committee of Jianghan University (No. JHDXLL2022-016). Every effort was made to minimize potential discomfort or distress experienced by the animals. All experiments used male C57 mice to eliminate confounding effects of the estrous cycle and to ensure consistent social defeat outcomes, as female C57 mice are not easily defeated by CD-1 mice [26].

### 2.2. AAV Vectors

The AAV vectors procured from WZ Biosciences Inc. (Jinan, China) were AAV9-U6-shRNA (Dnmt3a)-CMV-GFP (AAV-shDnmt3a-GFP), AAV9-CMV-GFP, AAV-EF1α-Dnmt3a-P2A-EGFP (AAV-Dnmt3a-GFP), AAV-EF1α-GFP, AAV-MBP-mir30-shRNA-GFP (AAV-MBP-shDnmt3a-GFP), and AAV-MBP-GFP.

### 2.3. Stereotaxic Surgical Procedures

Surgery was performed according to a previously described protocol [27]. Briefly, mice were anesthetized using isoflurane and securely positioned on a stereotaxic apparatus. For Dnmt3a knockdown, AAV-shDnmt3a-GFP or AAV-MBP-shDnmt3a-GFP was bilaterally infused into the NAc (350 nL; AP = +1.3 mm, ML= ± 0.7 mm, DV = −4.20 mm) at a rate of 100 nL/min using an R480 glass microelectrode syringe pump (RWD Life Technology Co., Ltd., Shenzhen, China). Similarly, for Dnmt3a overexpression, AAV-Dnmt3a-GFP was bilaterally injected into the NAc. Control groups received bilateral infusions of AAV-GFP or AAV-MBP-GFP. The virus was allowed to express for 3 weeks before behavioral testing. Injection sites were verified by fluorescence microscopy (Carl Zeiss Axio ImagerA2, Oberkochen, Germany). Animals with misplaced injections were excluded from the experimental groups.

### 2.4. Chronic Social Defeat Stress (CSDS)

CSDS is a well-established protocol that generates distinct cohorts of stress-susceptible mice (exhibiting depressive-like behaviors) and resilient individuals [28]. The CSDS procedure was conducted according to established methods [29]. Test mice were exposed daily to an unfamiliar, highly aggressive male CD1 mouse (with a latency to first attack of less than 1 min) for 5–10 min over up to 10 consecutive days. Following each physical interaction, the experimental mouse was transferred to a neighboring compartment within the same cage, allowing sensory contact without physical re-exposure, and maintained in this configuration for approximately 24 h. Control animals were housed in identical cages with non-aggressive conspecifics of the same strain. Twenty-four hours after the final social defeat session, all mice underwent sociability testing and were classified as either susceptible or resilient based on their SI scores.

### 2.5. Submaximal Social Defeat Stress (SSDS)

The SSDS paradigm is a well-established and rigorously validated method for investigating factors that influence stress vulnerability in mice. Following SSDS, control subjects typically exhibit normal SI behavior, whereas experimental manipulations that increase susceptibility result in pronounced social avoidance [30]. To explore conditions that may heighten sensitivity to social stress, we employed the SSDS protocol, subjecting mice to multiple rounds of aggressive encounters in a single day [31]. In each session, a test mouse was introduced into the home cage of a CD1 aggressor for a 5 min period, after which it was removed and allowed a 15 min rest interval. This sequence was carried out three times in total, with each exposure involving a novel CD1 aggressor. Upon completion of the last defeat episode, the stressed animals were transferred back to their home cages. Behavioral assessment using the SI test was performed the subsequent day.

### 2.6. Open Filed (OF) Test

Mice were permitted to freely explore a 50 × 50 × 50 cm^3^ milky white opaque enclosure. Each animal was gently placed in the center of the arena, oriented toward the wall, and its behavior was recorded for 10 min using an overhead camera. Locomotor activity and anxiety-related behaviors were analyzed using the Supermaze Video Analysis System V3.3 (Shanghai XinRuan Information Technology Co., Ltd., Shanghai, China), which automatically tracked total travel distance, time spent in the central zone, and the frequency of entries into this area. Reduced time in the center and fewer entries were interpreted as indicators of heightened anxiety.

### 2.7. SI Test

SI tests were performed 24 h after the last defeat, as described previously [27]. Mice were habituated in the testing rooms for 1 h before testing and all testing was performed under red-light conditions. SI tests were performed with mice freely exploring in a target-free arena (50 cm × 50 cm) for 5 min, followed by another 5  min target-present (CD-1) session during which target mice were confined in a cylindrical wire-mesh enclosure (10 cm × 15 cm). The Supermaze Video Analysis System V3.3 (Shanghai XinRuan Information Technology Co., Ltd.) was used to record the time spent in the interaction zone measuring 15 cm × 15 cm area. We calculated SI ratio as the ratio of time spent in the interaction zone with a CD-1 mouse present over time spent with the target absent. All mice with a SI ratio over 1 were classified as stress-resilient mice and all with a ratio below 1 as stress-susceptible mice.

### 2.8. Elevated Zero Maze (EZM)

The EZM apparatus consists of a circular runway elevated 100 cm above the ground, with an outer diameter of 45 cm and a width of 6 cm. The runway is divided into two open arms and two closed arms of equal length, arranged alternately. The closed arms are enclosed by inner and outer walls extending 10 cm above the runway surface to provide sheltered zones. At the beginning of the test, each mouse is placed in an open arm facing the center of the maze. Automated tracking and behavioral analysis are performed using the Supermaze Video Analysis System V3.3 (Shanghai Xinruan Information Technology Co., Ltd.), which records locomotor activity, time spent in the open and closed arms, and the number of entries into each zone over a 5 min period. Decreased time in the open arms and reduced entry frequency are interpreted as indicators of increased anxiety-like behavior in mice.

All behavioral tests in this study were conducted in a double-blinded manner. The specific blinding procedure was as follows: (1) Two independent experimenters were involved in the behavioral tests: one was responsible for grouping and labeling the animals (using random code numbers instead of group names) without participating in the behavioral detection; (2) the other experimenter, who was unaware of the grouping information and animal labels, performed all behavioral tests (including data collection and recording); (3) after the completion of all behavioral tests, the code was decoded by the first experimenter, and the data were matched with the corresponding groups for statistical analysis.

### 2.9. Western Blot

The NAc and mPFC of mice were rapidly dissected and mechanically homogenized in RIPA lysis buffer containing protease and phosphatase inhibitors (Beyotime Biotechnology, Shanghai, China) for total protein extraction. Protein concentrations were quantified using a BCA protein assay kit (Beyotime Biotechnology). Equal amounts of protein lysates were separated by 12% sodium dodecyl sulfate-polyacrylamide gel electrophoresis (SDS-PAGE) and transferred onto nitrocellulose membranes (Millipore, Darmstadt, Germany). Following three washes with phosphate-buffered saline (PBS), nonspecific binding sites were blocked by incubating the membranes in 10% skimmed milk at room temperature for 2 h. Membranes were then incubated overnight at 4 °C with the following primary antibodies: anti-mouse monoclonal antibodies β-actin (1:2000, ProteinTech, Wuhan, China), Dnmt3a, and Dnmt3b (1:1000, Santa Cruz, CA, USA); anti-rabbit polyclonal antibodies Dnmt1, MBP, D1 and D2 receptors (1:1000, Proteintech). After washing, membranes were incubated with horseradish peroxidase (HRP)-conjugated anti-mouse or anti-rabbit IgG secondary antibodies (1:5000, Beyotime Biotechnology) for 1 h at room temperature. Immunoreactive bands were detected using an ECL chemiluminescence reagent kit (Millipore), and band intensities were quantified using ImageJ 1.8.0 software (Montgomery, MD, USA).

### 2.10. Immunofluorescence

For histological analysis, mice were anesthetized with isoflurane and transcardially perfused before brain collection. Brains were post-fixed in 4% paraformaldehyde at 4 °C for 24 h. Coronal sections of the fixed brains were cut at a thickness of 40 μm and washed thoroughly in phosphate-buffered saline (PBS). Tissue slices were permeabilized with 0.3% Triton X-100 for 30 min and blocked with 5% normal goat serum (NGS) for 1 h at room temperature. Primary antibodies were applied overnight at 4 °C, including anti-mouse monoclonal Dnmt3a (1:250, Santa Cruz), anti-rabbit polyclonal NeuN (1:500, CST, Danvers, MA, USA), Iba1 (1:1000, Abcam, Boston, MA, USA), GFAP (1:500, CST), Olig2 (1:500, Abcam), NG2 (1:500, Abcam), and MBP (1:500, Proteintech). After PBS washes, sections were incubated with secondary antibodies (goat anti-mouse Alexa Fluor 488 and goat anti-rabbit Alexa Fluor 594, Thermo Fisher, Delaware, MA, USA) for 2 h at room temperature. Following additional PBS washes, tissue sections were mounted on glass slides and coverslipped using a mounting medium containing DAPI. Immunofluorescence images were captured using a laser scanning confocal microscope (Leica SP8, Wetzlar, Germany).

All virus-injected mice were verified post-experimentally for correct viral injection placement; animals with misplaced injections or undetectable transgene expression were excluded from the experimental groups. Briefly, mice were anesthetized and subjected to transcardial perfusion with cold PBS followed by 4% paraformaldehyde. Brains were then removed and post-fixed overnight in 4% paraformaldehyde, equilibrated in 30% sucrose solution until fully saturated, and sectioned coronally at 40 μm thickness. Tissue sections were washed and stained with DAPI. Fluorescent images were acquired using an Olympus BX51 FluoView microscope system (Olympus, Tokyo, Japan).

### 2.11. Statistical Analyses

Statistical analyses were performed using GraphPad Prism 8.0 software (San Diego, CA, USA). The normality of data distribution was evaluated using the Shapiro–Wilk normality test. Two-tailed unpaired *t*-tests were used to assess statistically significant differences between two groups in Western blot, SI, OF, and EZM experiments. One-way or two-way analysis of variance (ANOVA) was conducted followed by Bonferroni’s post hoc multiple comparisons test to examine the effects of social stress and Dnmt3a expression in the SI test, immunofluorescence, and Western blot analyses. Data are expressed as mean ± SEM, and statistical significance was defined as *p* < 0.05.

### 2.12. Clarification Regarding GenAI Tools

During the preparation of this work, the authors used Youdao Dictionary 11.3.4.0 (Beijing, China) for grammar checking and linguistic polishing. The authors have reviewed and edited the output and take full responsibility for the content of the publication.

## 3. Results

### 3.1. CSDS Induced an Increase in Dnmt3a Expression in the NAc of Stress-Susceptible Mice

**Figure 1:**
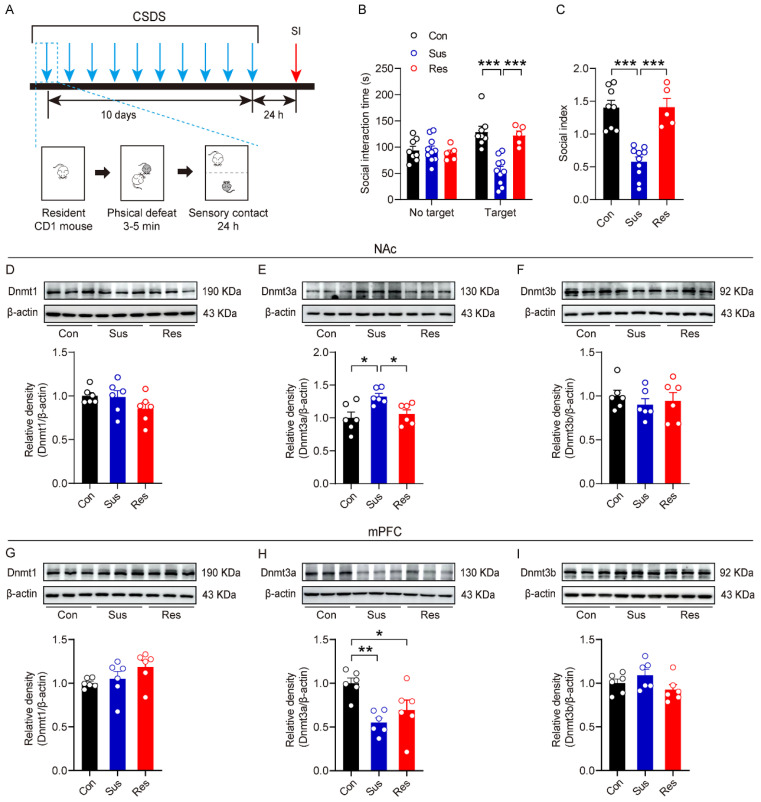
The effect of CSDS on the expression of Dnmt3a family members in the NAc of mice. (**A**) Experimental timeline and a detailed schematic of CSDS. (**B**) Summary plots of time spent in the SI zone in the presence or absence of a novel CD-1 target. Two-way ANOVA, target × stress interaction, F _(2, 40)_ = 13.6, *p* < 0.001; main effect of target, F _(1, 40)_ = 1.7, *p* = 0.199; main effect of stress, F _(2, 40)_ = 12.0, *p* < 0.001. Bonferroni post hoc test, Con target vs. Sus target, *p* < 0.001; Sus target vs. Res target, *p* < 0.001. (**C**) Summary plots of the social index. One-way ANOVA, F _(2,20)_ = 24.8, *p* < 0.001. Bonferroni post hoc test, Con vs. Sus, *p* < 0.001; Res vs. Sus, *p* < 0.001. (**D**,**G**) Summary plots of the expression of Dnmt1 in NAc (**D**) and mPFC (**G**). (**E**,**H**) Summary plots of the expression of Dnmt3a in NAc (**E**) and mPFC (**H**). One-way ANOVA, F _(2,15)_ = 6.3, *p*= 0.01. Bonferroni post hoc test, Con vs. Sus, *p* = 0.012; Res vs. Sus, *p* = 0.038 (NAc); One-way ANOVA, F _(2,15)_ = 8.19, *p* = 0.004. Bonferroni post hoc test, Con vs. Sus, *p* = 0.003; Con vs. Res, *p* = 0.041 (mPFC). (**F**,**I**) Summary plots of the expression of Dnmt3b in NAc (**F**) and mPFC (**I**). For (**B**,**C**), n = 8 Con, 10 Sus, 5 Res. For (**D**–**I**), n = 6 per group. * *p* < 0.05; ** *p* < 0.01; *** *p* < 0.001. CSDS, chronic social defeat stress; NAc, nucleus accumbens; mPFC, medial prefrontal cortex; SI, social interaction. The original WB images are shown in Figure S1.

To investigate the potential role of Dnmt in stress susceptibility, we first established a CSDS model in mice and assessed behavioral phenotypes using SI test (Figure 1A). As shown in Figure 1B,C, based on the SI ratio, mice were stratified into three groups: compared with unstressed controls, the stress-susceptible (Sus) group exhibited significant social avoidance (SI ratio < 1), whereas the stress-resistant (Res) group displayed preserved social preference (SI ratio ≥ 1). We then examined protein expression levels of Dnmt1, Dnmt3a, and Dnmt3b in the NAc and mPFC via Western blotting. In the NAc, Dnmt3a expression was significantly elevated in the Sus group relative to both the control and Res groups (Figure 1E). This effect was highly specific, as no significant differences were observed in NAc expression of Dnmt1 (Figure 1D) or Dnmt3b (Figure 1F) across groups. In contrast, in the mPFC, Dnmt3a expression was significantly reduced in both Sus and Res groups compared to controls (Figure 1H), with no significant difference between the Sus and Res groups. Furthermore, consistent with NAc findings, expression levels of Dnmt1 (Figure 1G) and Dnmt3b (Figure 1I) in the mPFC remained unchanged. These results demonstrated that CSDS induces region- and isoform-specific alterations in Dnmt expression. The selective upregulation of Dnmt3a in the NAc is specifically associated with social stress susceptibility, while its downregulation in the mPFC appears to reflect a generalized stress response. Collectively, these findings indicated that NAc-localized Dnmt3a may serve as a key regulatory molecule selectively involved in modulating individual vulnerability to social stress.

### 3.2. Overexpression of Dnmt3a in the NAc Enhanced Susceptibility to Social Stress

**Figure 2:**
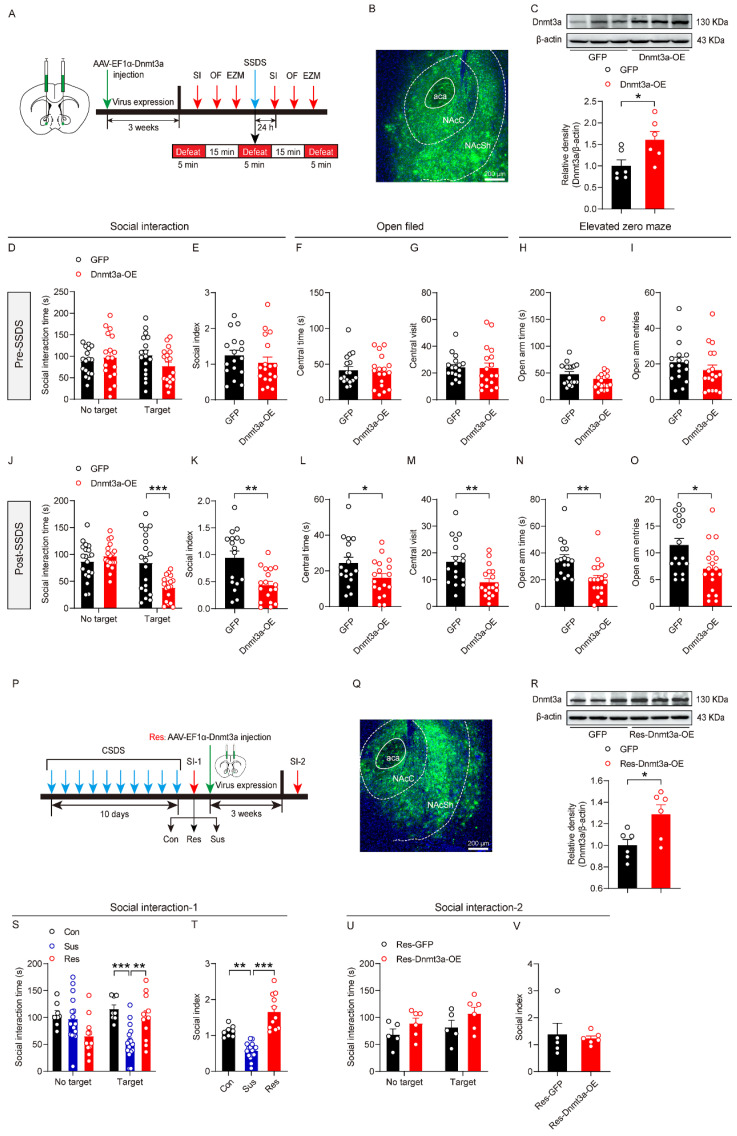
The effect of overexpression of Dnmt3a in the NAc on susceptibility to social stress. (**A**) Schematic representation of the experimental procedure. (**B**) Fluorescent localization of the virus injection site. (**C**) Western blot analysis of Dnmt3a expression in the NAc. Unpaired two-tailed *t*-test, t _(10)_ = 2.51, *p* = 0.031. (**D**) Summary plots of time spent in the SI zone in the presence or absence of a novel CD-1 target before SSDS modeling. (**E**) Summary plots of the social index before SSDS modeling. (**F**,**G**) Summary plots of time spent and number of entries in the middle area of the open field before SSDS modeling. (**H**,**I**) Summary plots of time spent and number of entries in the open arms of EZM before SSDS modeling. (**J**) Summary plots of time spent in the SI zone in the presence or absence of a novel CD-1 target after SSDS modeling. Two-way ANOVA, target × gene interaction, F _(1, 66)_ = 9.09, *p* = 0.004; main effect of target, F _(1, 66)_ = 15.2, *p* < 0.001; main effect of gene, F _(1, 66)_ = 3.34, *p* = 0.072. Bonferroni post hoc test, GFP target vs. Dnmt3a-OE target, *p* = 0.02. (**K**) Summary plots of the social index after SSDS modeling. Unpaired two-tailed *t*-test, t _(33)_ = 3.54, *p* = 0.001. (**L**,**M**) Summary plots of time spent and number of entries in the middle area of the open field after SSDS modeling. Unpaired two-tailed *t*-test for (**L**), t _(33)_ = 2.07, *p* = 0.046; unpaired two-tailed t-test for (**M**), t _(33)_ = 3.17, *p* = 0.003. (**N**,**O**) Summary plots of time spent and entries in the open arms of EZM after SSDS modeling. Unpaired two-tailed *t*-test for (**N**), t _(33)_ = 3.43, *p* = 0.002; unpaired two-tailed t-test for (**O**), t _(33)_ = 2.73, *p* = 0.01. (**P**) Schematic representation of the experimental design for Dnmt3a overexpression in the Res group. (**Q**) Fluorescence imaging of virus infection in the NAc of Res group. (**R**) Western blot validation of Dnmt3a expression in Res group. Unpaired two-tailed *t*-test, t _(10)_ = 2.75, *p* = 0.02. (**S**) Summary plots of time spent in the SI zone in the presence or absence of a novel CD-1 target. Two-way ANOVA, target × stress interaction, F _(2, 64)_ = 8.4, *p* < 0.001; main effect of target, F _(1, 64)_ = 0.005, *p* = 0.942; main effect of stress, F _(2, 64)_ = 5.34, *p* = 0.007. Bonferroni post hoc test, Con target vs. Sus target, *p* < 0.001; Sus target vs. Res target, *p* = 0.005. (**T**) Summary plots of the social index. One-way ANOVA, F _(2,32)_ = 36.5, *p* < 0.001. Bonferroni post hoc test, Con vs. Sus, *p* = 0.001; Res vs. Sus, *p* < 0.001. (**U**) Summary plots of SI time following Dnmt3a overexpression in the Res group. (**V**) Summary plots of the social index following Dnmt3a overexpression in the Res group. For (**C**,**R**), n = 6 per group. For (**D**–**O**), n = 17 GFP, 18 Dnmt3a-OE. For (**S**,**T**), n = 8 Con, 16 Sus, 11 Res. For (**U**,**V**), n = 5 Res-GFP, 6 Res-Dnmt3a-OE. * *p* < 0.05; ** *p* < 0.01; *** *p* < 0.001. SSDS, submaximal social defeat stress. The original WB images are shown in Figure S2.

To investigate the regulatory role of Dnmt3a in the NAc in modulating susceptibility to social stress in mice, we stereotaxically injected a recombinant AAV-expressing Dnmt3a (AAV-EF1α-Dnmt3a, Dnmt3a-OE group) into the NAc to specifically upregulate Dnmt3a expression in this brain region, thereby establishing a mouse model of Dnmt3a overexpression for assessing stress susceptibility. We aimed to determine whether elevated Dnmt3a levels in the NAc are a critical factor in driving the emergence of a stress-susceptible phenotype (Figure 2A). Control mice received injections of an empty vector encoding green fluorescent protein (AAV-EF1α-GFP, GFP group). Three weeks post-injection, fluorescence microscopy revealed robust and localized green fluorescence signals in both the NAc core (NAcC) and shell (NAcSh) (Figure 2B), confirming successful and spatially restricted viral transduction of the target region. Western blot analysis further demonstrated that Dnmt3a protein expression in the NAc was significantly higher in the Dnmt3a-OE group compared to the GFP control group (Figure 2C), validating efficient and specific overexpression of Dnmt3a in the NAc. Next, we implemented a modified CSDS paradigm, termed SSDS in the manuscript, to assess early behavioral responses; in which animals exposed to social stress did not initially exhibit deficits in the SI. We then evaluated SI and anxiety-related behaviors before and after SSDS establishment. Behavioral assessments showed that prior to SSDS exposure, Dnmt3a overexpression had no significant effect on baseline social or anxiety-like behaviors (Figure 2D–I). However, following SSDS, Dnmt3a-OE mice displayed markedly increased social avoidance and heightened anxiety levels compared to controls (Figure 2J–O). These findings indicated that NAc-specific Dnmt3a overexpression significantly enhanced susceptibility to social stress without altering baseline behavior in unstressed mice. To further examine whether Dnmt3a overexpression can convert stress-resilient individuals into a susceptible state, we delivered the Dnmt3a-overexpressing virus into the NAc of stress-resilient mice identified through CSDS (Figure 2P). After 3 weeks of viral expression, fluorescence microscopy revealed widespread green fluorescence signals in the NAc, indicating successful viral transduction in the target brain region (Figure 2Q). Western blot analysis further showed that Dnmt3a protein expression in the NAc was significantly higher in the Dnmt3a-OE group than in the GFP control group (Figure 2R), confirming efficient and specific overexpression of Dnmt3a in the NAc. Figure 2S,T confirm that CSDS effectively induces phenotypic differentiation into “susceptible” and “resistant” subpopulations in mice, thereby enabling the selection of qualified subjects for the subsequent intervention experiment in the Res group. Figure 2U,V show that, compared with GFP controls, Dnmt3a overexpression does not alter SI time or SI index in resilient mice, suggesting that elevated Dnmt3a levels in the NAc are insufficient to override inherent stress resilience.

### 3.3. Downregulation of Dnmt3a in the NAc Reduced Susceptibility to Social Stress

**Figure 3:**
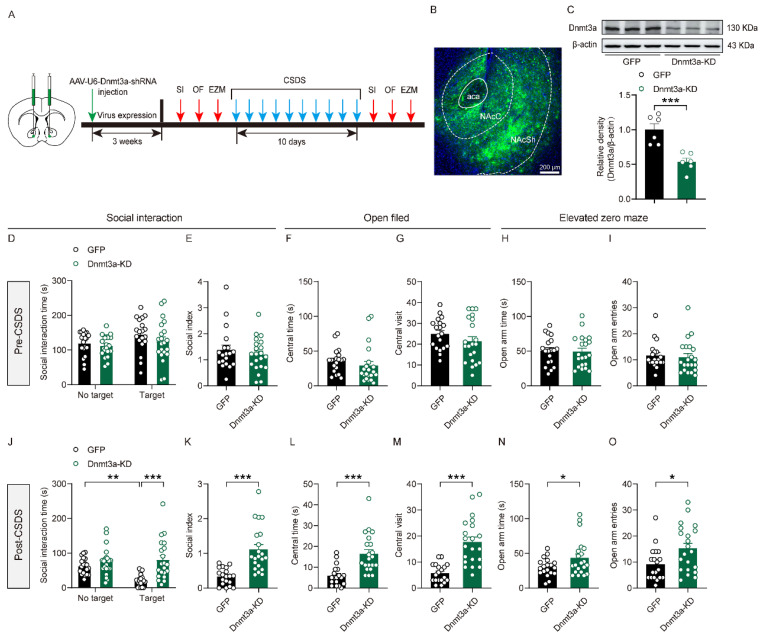
The effect of downregulation of Dnmt3a in the NAc on susceptibility to social stress. (**A**) Schematic representation of the experimental procedure. (**B**) Fluorescent localization of the virus injection site. (**C**) Western blot analysis of Dnmt3a expression in the NAc. Unpaired two-tailed *t*-test, t _(10)_ = 4.67, *p* < 0.001. (**D**) Summary plots of time spent in the SI zone in the presence or absence of a novel CD-1 target before CSDS modeling. (**E**) Summary plots of the social index before CSDS modeling. (**F**,**G**) Summary plots of time spent and number of entries in the middle area of the open field before CSDS modeling. (**H**,**I**) Summary plots of time spent and number of entries in the open arms of EZM before CSDS modeling. (**J**) Summary plots of time spent in the SI zone in the presence or absence of a novel CD-1 target after CSDS modeling. Two-way ANOVA, target × gene interaction, F _(1, 76)_ = 6.88, *p* = 0.011; main effect of target, F _(1, 76)_ = 4.21, *p* = 0.044; main effect of gene, F _(1, 76)_ = 17.3, *p* < 0.001. Bonferroni post hoc test, GFP no target vs. GFP target, *p* = 0.004; GFP target vs. Dnmt3a-KD target, *p* < 0.001. (**K**) Summary plots of the social index after CSDS modeling. Unpaired two-tailed *t*-test, t _(38)_ = 4.88, *p* < 0.001. (**L**,**M**) Summary plots of time spent and number of entries in the middle area of the open field after CSDS modeling. Unpaired two-tailed *t*-test for (**L**), t _(38)_ = 4.42, *p* < 0.001; unpaired two-tailed *t*-test for (**M**), t _(38)_ = 5.54, *p* < 0.001. (**N**,**O**) Summary plots of time spent and entries in the open arms of EZM after CSDS modeling. Unpaired two-tailed *t*-test for (**N**), t _(38)_ = 2.03, *p* = 0.05; unpaired two-tailed *t*-test for (**O**), t _(38)_ = 2.48, *p* = 0.018. For (**C**), n = 6 per group. For (**D**–**O**), n = 19 GFP, 21 Dnmt3a-KD. * *p* < 0.05; ** *p* < 0.01; *** *p* < 0.001. The original WB images are shown in Figure S3.

We further employed the CSDS model to investigate the effects of Dnmt3a downregulation on the susceptibility of mice to social stress. Dnmt3a knockdown virus was stereotaxically injected into the NAc of mice to specifically reduce Dnmt3a expression in this brain region (Figure 3A). Three weeks after viral expression, fluorescence microscopy revealed widespread green fluorescent signals in both the core (NAcC) and shell (NAcSh) subregions of the NAc (Figure 3B), indicating successful viral transduction; Western blot analysis further confirmed that Dnmt3a protein expression in the NAc was significantly reduced compared with the GFP control group (Figure 3C), validating the efficiency of gene knockdown. Subsequently, a CSDS model was established in mice, and SI and anxiety-related behaviors were assessed before and after stress exposure. Results showed that prior to CSDS, NAc-specific Dnmt3a knockdown did not significantly affect baseline SI or anxiety-like behaviors (Figure 3D–I). However, following stress exposure, mice in the Dnmt3a knockdown group exhibited significantly reduced social avoidance and lower anxiety levels compared to controls (Figure 3J–O). These findings indicated that downregulation of Dnmt3a expression in the NAc markedly decreases susceptibility to social stress. These data established a critical role for NAc Dnmt3a in regulating behavioral responses to social stress.

### 3.4. Dnmt3a in the NAc Is Predominantly Localized and Expressed in Olig2-Positive Cells

**Figure 4:**
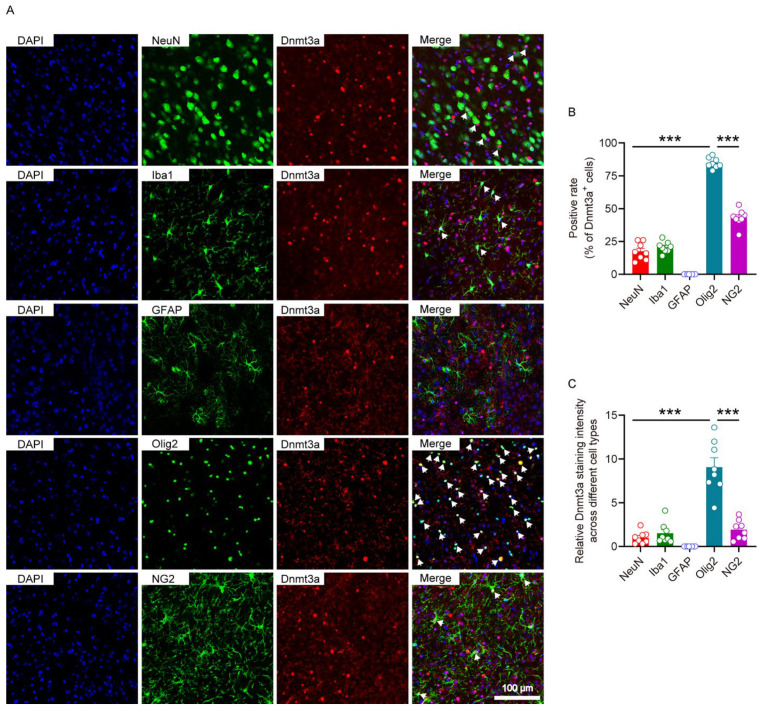
The distribution and expression of Dnmt3a in distinct cell types in the NAc. (**A**) Immunofluorescence co-localization of Dnmt3a with cell type-specific markers in the NAc. (**B**) Proportion of Dnmt3a-positive cells among distinct cell types in the NAc. One-way ANOVA, F _(4,35)_ = 358, *p* < 0.001. Bonferroni post hoc test, Olig2 vs. NeuN, Iba1, GFAP, NG2, *p* < 0.001. (**C**) Relative immunofluorescence intensity of Dnmt3a across distinct cell types in the NAc. One-way ANOVA, F _(4,35)_ = 43.8, *p* < 0.001. Bonferroni post hoc test, Olig2 vs. NeuN, Iba1, GFAP, NG2, *p* < 0.001. n = 8 per group. *** *p* < 0.001.

We performed immunofluorescence double-labeling to co-stain Dnmt3a with cell type-specific markers for neurons (NeuN), microglia (Iba1), astrocytes (GFAP), oligodendrocytes (Olig2), and oligodendrocyte precursor cells (NG2) to determine the distribution and expression pattern of Dnmt3a across distinct cell populations in the NAc (Figure 4A). The results revealed that the co-localization rate and Dnmt3a expression level in oligodendrocytes were significantly higher compared to other cell types, while expression in oligodendrocyte precursor cells was markedly lower (Figure 4B,C). These findings suggest that Dnmt3a may exert its regulatory role in social stress susceptibility primarily within mature oligodendrocytes.

### 3.5. CSDS Induced an Increase in Dnmt3a Expression in Olig2-Positive Cells and Reduced Myelination and Dopamine D1 Receptor Expression

**Figure 5:**
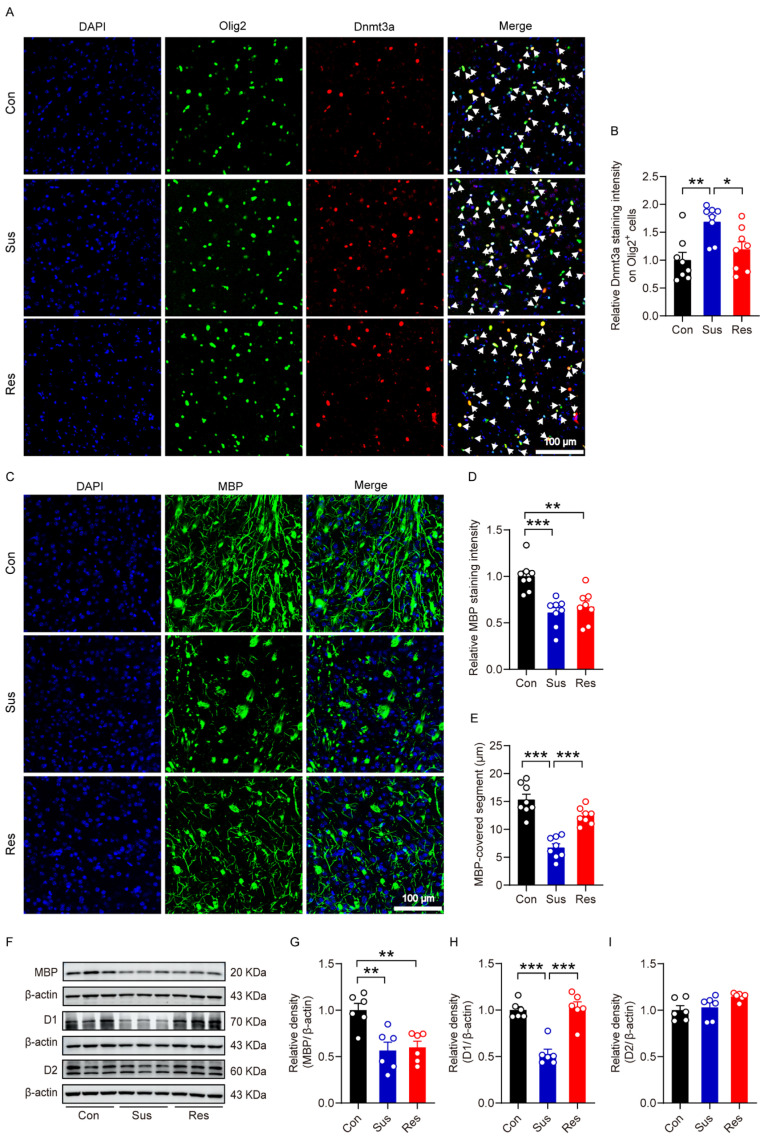
The effect of CSDS on Dnmt3a expression, myelin formation, and dopamine receptor expression in the NAc. (**A**) Immunofluorescence co-localization of Olig2^+^ cells with Dnmt3a in the NAc. (**B**) Quantification of the relative immunofluorescence intensity of Dnmt3a in Olig2^+^ cells. One-way ANOVA, F _(2,21)_ = 7.53, *p* = 0.003. Bonferroni post hoc test, Con vs. Sus, *p* = 0.003; Res vs. Sus, *p* = 0.034. (**C**) Immunofluorescence staining of MBP in the NAc. (**D**) Quantification of the relative MBP immunofluorescence intensity. One-way ANOVA, F _(2,21)_ = 13.0, *p* < 0.001. Bonferroni post hoc test, Con vs. Sus, *p* < 0.001; Con vs. Res, *p* = 0.002. (**E**) Quantification of the length of MBP-positive segments. One-way ANOVA, F _(2,21)_ = 34.1 *p* < 0.001. Bonferroni post hoc test, Con vs. Sus, *p* < 0.001; Res vs. Sus, *p* < 0.001. (**F**) The protein expression bands of MBP, dopamine D1 and D2 receptors in the NAc. (**G**) Summary plots of the expression of MBP. One-way ANOVA, F _(2,15)_ = 10.1, *p* = 0.002. Bonferroni post hoc test, Con vs. Sus, *p* = 0.003; Con vs. Res, *p* = 0.006. (**H**) Summary plots of the expression of dopamine D1 receptor. One-way ANOVA, F _(2,15)_ = 28.4, *p* < 0.001. Bonferroni post hoc test, Con vs. Sus, *p* < 0.001; Res vs. Sus, *p* < 0.001. (**I**) Summary plots of the expression of dopamine D2 receptor. For (**A**–**E**), n = 8 per group. For (**F**–**I**), n =6 per group. * *p* < 0.05; ** *p* < 0.01; *** *p* < 0.001. The original WB images are shown in Figure S4.

To investigate whether Dnmt3a is differentially expressed in oligodendrocytes of the nucleus accumbens (NAc) following CSDS, we performed immunofluorescence staining. The results revealed that Dnmt3a fluorescence intensity in the NAc was significantly elevated in susceptible mice compared to both control and resilient mice (Figure 5A,B), indicating that upregulation of Dnmt3a in oligodendrocytes may serve as a key molecular mechanism underlying susceptibility to social stress. To further assess myelin integrity, we examined the expression and morphology of MBP in the NAc using immunofluorescence. While MBP fluorescence intensity was significantly reduced in both susceptible and resilient mice relative to control mice, with no significant difference between Sus and Res groups, the length of myelinated segments was markedly shorter in the Sus group compared to both Con and Res groups (Figure 5C–E). This suggests that susceptible mice exhibit specific structural deficits in myelination, independent of overall MBP expression levels. These findings imply that localized myelin structural impairment, particularly shortened myelinated segments, may constitute a critical pathological feature associated with susceptibility to social stress. Consistent with immunofluorescence data, Western blot analysis confirmed a significant downregulation of MBP in the NAc of both Sus and Res mice compared to controls (Figure 5F,G). Additionally, to evaluate dopaminergic signaling alterations, we assessed the expression levels of dopamine D1 and D2 receptors in the NAc. Western blot results demonstrated that D1 receptor expression was significantly lower in the NAc of susceptible mice than in control and resilient mice, whereas D2 receptor levels remained unchanged across all three groups (Figure 5F,H,I). These data suggest that downregulation of D1 receptors in the NAc is specifically linked to stress susceptibility, while D2 receptor expression does not appear to contribute to this behavioral phenotype.

### 3.6. Dnmt3a in the NAc Negatively Regulated the Expression of MBP and Dopamine D1 Receptor

**Figure 6:**
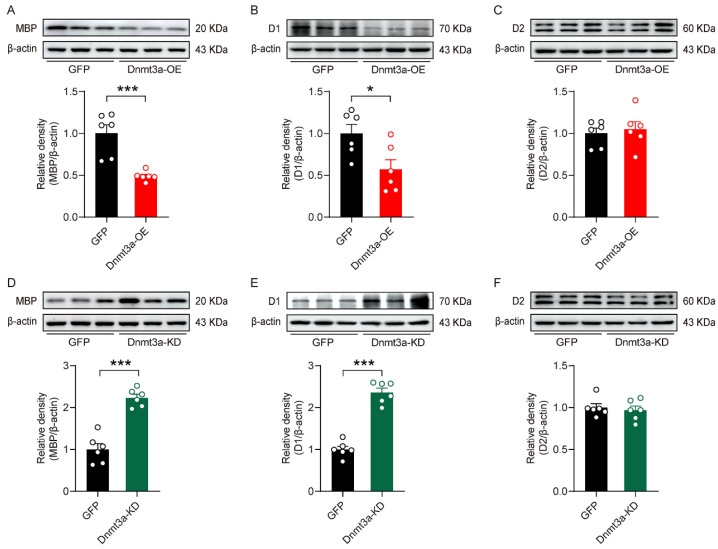
The regulatory role of Dnmt3a in the NAc on MBP and D1 receptor expression. (**A,D**) Summary plots of MBP expression in mice with Dnmt3a overexpression and knockdown in the NAc. Unpaired two-tailed *t*-test for (**A**), t _(10)_ = 4.84, *p* < 0.001; unpaired two-tailed *t*-test for (**D**), t _(10)_ = 7.62, *p* < 0.001. (**B**,**E**) Summary plots of dopamine D1 receptor expression in mice with Dnmt3a overexpression and knockdown in the NAc. Unpaired two-tailed *t*-test for (**B**), t _(10)_ = 2.74, *p* = 0.021; unpaired two-tailed *t*-test for (**E**), t _(10)_ = 10.5, *p* < 0.001. (**C**,**F**) Summary plots of dopamine D2 receptor expression in mice with Dnmt3a overexpression and knockdown in the NAc. Unpaired two-tailed *t*-test for (**C**), t _(10)_ = 0.451, *p* = 0.662; unpaired two-tailed *t*-test for (**F**), t _(10)_ = 0.483, *p* = 0.639. n = 6 per group. * *p* < 0.05; *** *p* < 0.001. The original WB images are shown in Figure S5.

To investigate the role of Dnmt3a in myelin-related protein expression and dopamine receptor signaling, we performed Western blot analysis. Our results demonstrated that Dnmt3a overexpression significantly suppressed the expression of MBP and D1 receptor (Figure 6A,B), whereas knockdown of Dnmt3a enhanced their expression (Figure 6D,E). These findings provided consistent evidence from both gain-of-function and loss-of-function approaches that Dnmt3a exerts a negative regulatory effect on MBP and D1 receptor expression. In contrast, Dnmt3a expression levels did not significantly alter D2 receptor protein levels (Figure 6C,F). This selective regulation likely arises from the presence of Dnmt3a-mediated DNA methylation sites in the promoter regions of the MBP and D1 receptor genes, whereas the D2 receptor gene promoter lacks such sites or is epigenetically insensitive to this modification.

### 3.7. Knockdown of Dnmt3a Expression in Olig2-Positive Cells of the NAc Reduced Susceptibility to Social Stress

**Figure 7:**
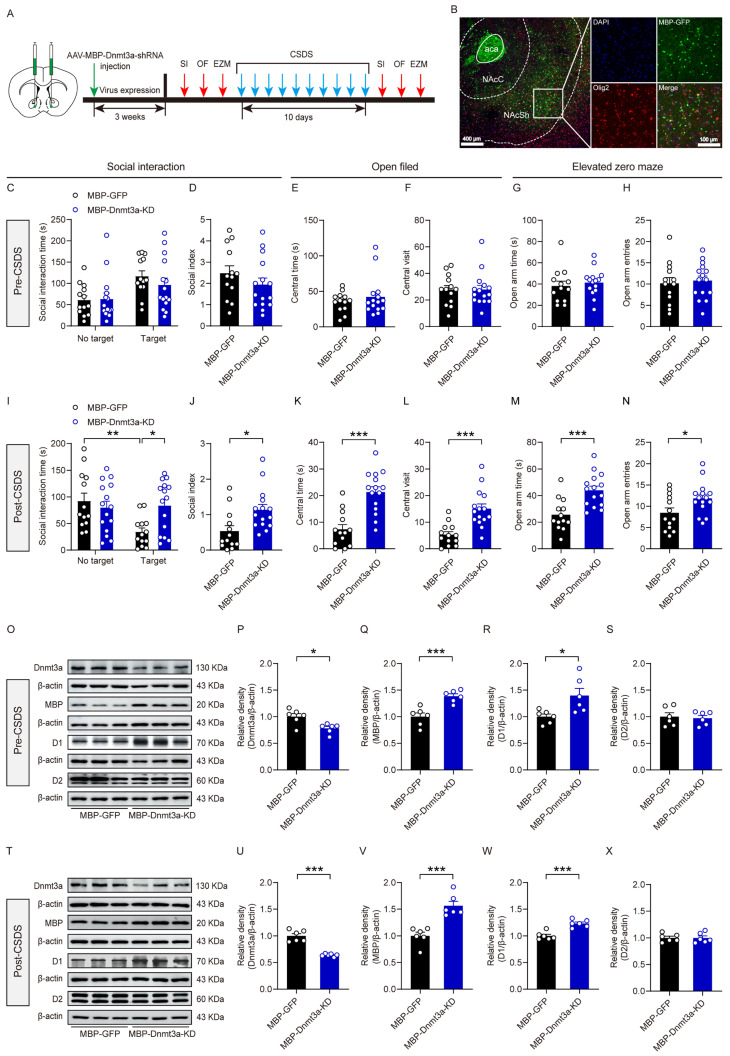
The effect of Dnmt3a knockdown in NAc oligodendrocytes on susceptibility to social stress. (**A**) Schematic diagram of the experimental procedure. (**B**) Fluorescent localization of the virus injection site. (**C**) Summary plots of time spent in the SI zone in the presence or absence of a novel CD-1 target before CSDS modeling. (**D**) Summary plots of the social index before CSDS modeling. (**E**,**F**) Summary plots of time spent and number of entries in the middle area of the open field before CSDS modeling. (**G**,**H**) Summary plots of time spent and number of entries in the open arms of EZM before CSDS modeling. (**I**) Summary plots of time spent in the SI zone in the presence or absence of a novel CD-1 target after CSDS modeling. Two-way ANOVA, target × gene interaction, F _(1, 52)_ = 6.58, *p* = 0.013; main effect of target, F _(1, 52)_ = 5.06, *p* = 0.029; main effect of gene, F _(1, 52)_ = 2.37, *p* = 0.13. Bonferroni post hoc test, MBP-GFP no target vs. MBP-GFP target, *p* = 0.011; MBP-GFP target vs. MBP-Dnmt3a-KD target, *p* = 0.004. (**J**) Summary plots of the social index after CSDS modeling. Unpaired two-tailed *t*-test, t _(26)_ = 2.77, *p* = 0.01. (**K**,**L**) Summary plots of time spent and number of entries in the middle area of the open field after CSDS modeling. Unpaired two-tailed *t*-test for (**K**), t _(26)_ = 5.11, *p* < 0.001; unpaired two-tailed *t*-test for (**L**), t _(26)_ = 4.41, *p* < 0.001. (**M**,**N**) Summary plots of time spent and entries in the open arms of EZM after CSDS modeling. Unpaired two-tailed *t*-test for (**M**), t _(26)_ = 3.99, *p* < 0.001; unpaired two-tailed *t*-test for (**N**), t _(26)_ = 2.28, *p* = 0.031. (**O**,**T**) The protein expression bands of Dnmt3a, MBP, dopamine D1 and D2 receptors in the NAc. (**P**,**U**) Summary plots of the expression of Dnmt3a. Unpaired two-tailed *t*-test for (**P**), t _(10)_ = 3.13, *p* = 0.011; unpaired two-tailed *t*-test for (**U**), t _(10)_ = 7.99, *p* < 0.001. (**Q**,**V**) Summary plots of the expression of MBP. Unpaired two-tailed *t*-test for (**Q**), t _(10)_ = 4.59, *p* < 0.001; unpaired two-tailed *t*-test for (**V**), t _(10)_ = 5.19, *p* < 0.001. (**R**,**W**) Summary plots of the expression of dopamine D1 receptor. Unpaired two-tailed t-test for (**R**), t _(10)_ = 2.78, *p* = 0.02; unpaired two-tailed *t*-test for (**W**), t _(10)_ = 5.64, *p* < 0.001. (**S**,**X**) Summary plots of the expression of dopamine D2 receptor. Unpaired two-tailed *t*-test for (**S**), t _(10)_ = 0.0548, *p* = 0.957; unpaired two-tailed *t*-test for (**X**), t _(10)_ = 0.321, *p* = 0.755. For (**C**–**N**), n = 13 MBP-GFP, 15 MBP-Dnmt3a-KD. For (**O**–**X**), n = 6 per group. * *p* < 0.05; ** *p* < 0.01; *** *p* < 0.001. The original WB images are shown in Figure S6.

To further investigate the role of Dnmt3a in oligodendrocytes in regulating social stress susceptibility, we injected an oligodendrocyte-specific Dnmt3a knockdown virus into the NAc of mice to selectively downregulate Dnmt3a expression in oligodendrocytes within this brain region. Three weeks post-injection, we first assessed baseline social behavior and anxiety-related phenotypes, then established a CSDS model, and subsequently re-evaluated the same behavioral parameters after model induction (Figure 7A). Three weeks after viral expression, fluorescence microscopy revealed widespread green fluorescent signals in both NAcC and NAcSh subregions of the NAc (Figure 7B), indicating successful viral transduction in the target region. Behavioral results revealed that prior to CSDS exposure, knockdown of Dnmt3a in NAc oligodendrocytes did not significantly alter SI or anxiety levels (Figure 7C–H); however, following CSDS, Dnmt3a knockdown markedly alleviated social avoidance and reduced anxiety-like behaviors (Figure 7I–N). These findings demonstrated that downregulation of Dnmt3a in NAc oligodendrocytes significantly reduces susceptibility to social stress. Western blot analysis showed that, before CSDS modeling, AAV-MBP-Dnmt3a-shRNA significantly decreased Dnmt3a expression while increasing MBP and D1 receptor levels compared to control mice, with no significant change in D2 receptor expression (Figure 7O–S). After CSDS, the virus continued to effectively suppress Dnmt3a and sustainably upregulate MBP and D1 receptor expression, whereas D2 receptor levels remained unchanged (Figure 7T–X). Taken together, these data indicated that oligodendrocyte-specific Dnmt3a expression in the NAc plays a critical role in modulating social stress susceptibility.

## 4. Discussion

This study, through systematic animal experiments and molecular biological analyses, has elucidated the pivotal role of Dnmt3a in NAc oligodendrocytes in regulating susceptibility to social stress, revealing a novel regulatory axis, the Dnmt3a-MBP/D1 receptor-NAc neural circuit. CSDS was found to specifically upregulate Dnmt3a in NAc oligodendrocytes, which in turn suppresses the expression of MBP and dopamine D1 receptors, thereby compromising myelin integrity and attenuating dopaminergic signaling. These cellular and molecular alterations ultimately lead to dysfunction in social-related neural circuits, manifesting as social avoidance and anxiety-like behaviors. By demonstrating glial cell involvement in behavioral regulation, this study challenges the neuron-centric paradigm in psychiatric research and provides compelling evidence for a glia-neuron interactive mechanism underlying social stress-related disorders. Furthermore, it established Dnmt3a in the NAc as a promising therapeutic target, offering theoretical support for future translational research and clinical applications.

The present study demonstrated that CSDS induces highly brain region- and subtype-specific alterations in Dnmt3a expression. In the NAc, only Dnmt3a, not Dnmt1 or Dnmt3b, was upregulated in stress-susceptible mice; no change occurred in resilient mice. In the mPFC, Dnmt3a was downregulated after stress in both susceptible and resilient mice, indicating a general stress response rather than a marker of susceptibility. Thus, Dnmt3a plays opposite, region-specific roles: in the NAc, it signals vulnerability to social stress; in the mPFC, it supports broad stress adaptation. Furthermore, the specificity of Dnmt3a’s role is consistent with known functional distinctions among Dnmts: Dnmt3a mediates de novo DNA methylation and is uniquely poised to drive activity-dependent, long-term transcriptional changes in response to environmental challenges, whereas Dnmt1 maintains pre-existing methylation patterns and Dnmt3b exhibits low and restricted expression in the adult brain [32]. Thus, the stress-susceptibility-specific upregulation of Dnmt3a in the NAc may drive persistent epigenetic remodeling of target genes, leading to enduring alterations in neural circuit function and ultimately shaping individual differences in behavioral responses to stress.

We further employed gain- and loss-of-function approaches to conclusively demonstrate the dual regulatory function of NAc Dnmt3a in modulating social stress susceptibility: overexpression of Dnmt3a increased susceptibility, whereas knockdown of its expression conferred resilience. These findings are consistent with prior evidence showing that Dnmt3a overexpression in the NAc promotes social avoidance in stressed mice, while pharmacological inhibition of Dnmt activity ameliorates CSDS-induced behavioral deficits [33]. Notably, the behavioral effects of Dnmt3a manipulation exhibit strict boundary conditions: altered Dnmt3a expression affects social behavior only in mice exposed to social stress, with no significant changes observed in baseline sociability or anxiety-like behaviors in unstressed controls. This indicated that Dnmt3a does not govern fundamental social functioning per se, but instead modulates the neural sensitivity to social stress, thereby shaping the stress response phenotype. Importantly, Dnmt3a overexpression failed to override the resilient phenotype, suggesting that stress resistance is maintained by robust, potentially homeostatic molecular mechanisms that cannot be easily disrupted by single-gene perturbations. This finding underscored a critical conceptual advance: while Dnmt3a serves as a key regulatory node in promoting stress susceptibility, it is not the sole determinant of individual outcomes. Rather, the balance between susceptibility and resilience emerges from the coordinated action of multiple molecular pathways. Future studies should therefore focus on characterizing the molecular signature of resilience and elucidating the interactive network between Dnmt3a and other epigenetic, transcriptional, and circuit-level regulators to fully understand the mechanistic basis of individual differences in stress vulnerability.

Oligodendrocytes are primarily responsible for the formation and maintenance of myelin, which serves as an “insulating layer” essential for efficient neural signal transmission; the structural and functional integrity of myelin directly governs the conduction efficiency of neural circuits [34,35]. In this study, Dnmt3a in the NAc was predominantly localized to oligodendrocytes, and oligodendrocyte-specific knockdown of Dnmt3a significantly reduced social stress susceptibility in mice, consistent with the effects observed in global knockdown experiments. This finding introduced a novel “glia-neuron interaction” framework for understanding the pathophysiology of mental disorders. Prior evidence has demonstrated that neuron–non-neuronal cell coupling networks contribute to chronic stress-induced electrophysiological alterations in lateral habenula neurons [36], providing a conceptual foundation for glial involvement in modulating stress-related circuit dynamics. Notably, in stress-susceptible mice, the NAc exhibited not only reduced expression of MBP, but also significant structural deficits, including shortened myelinated internodes, indicating that myelin dysfunction constitutes a key pathological substrate underlying social stress susceptibility. This is consistent with prior work reporting stress-induced MBP downregulation in the NAc of both susceptible and resilient mice; however, our data reveal that only susceptible animals display concomitant ultrastructural abnormalities, suggesting that MBP loss alone is insufficient to confer susceptibility, whereas its combination with structural disorganization may be pathognomonic. It is important to highlight that those studies found no significant change in myelin segment length across stressed animals compared to controls [37]. The discrepancy may arise from differences in experimental paradigms: our CSDS protocol employed longer daily agonistic interactions (5–10 min/day), compared to the standard 5 min/day used in prior work. This higher-intensity CSDS regimen may induce myelin damage in susceptible individuals that exceeds compensatory capacity, leading to measurable structural shortening of myelinated segments. Given the negative regulatory effect of Dnmt3a on MBP expression, we proposed that oligodendrocytes modulate myelin formation and stability through Dnmt3a-mediated epigenetic mechanisms, thereby influencing the functional integrity of NAc-dependent social circuits and ultimately shaping individual stress vulnerability. This mechanism expands the traditional view of glial cells by demonstrating that oligodendrocytes actively regulate emotional and social behaviors via myelin plasticity, rather than serving merely as passive structural support. Reduced MBP expression and shortened internodal length impair axonal conduction velocity and disrupt neural synchrony, compromising circuit-level communication [38]. However, the mechanistic link between Dnmt3a and transcriptional repression of MBP remains largely correlative. Notably, a mechanistic study in Parkinson’s disease has experimentally validated the functional relationship between Dnmt3a and MBP: it demonstrated that Dnmt3a indirectly suppresses MBP expression by downregulating signal transducer and activator of transcription 5B (STAT5B), which is consistent with our results [39]. Concurrently, dopamine D1 receptors, primarily expressed on medium spiny neurons in the NAc, are critical for reward processing and social motivation [40]; their downregulation attenuates dopaminergic signaling, reduces sensitivity to social rewards, and promotes social avoidance [41]. Importantly, this study revealed that Dnmt3a selectively regulates D1 receptor expression without affecting D2 receptors, aligning with the distinct roles of these receptor subtypes in behavior: D1 receptor signaling drives approach-related behaviors such as social engagement and reward seeking, whereas D2 receptor pathways are more closely associated with behavioral inhibition and conflict monitoring [42,43]. Thus, Dnmt3a orchestrates a dual-pathway disruption of NAc circuit function by impairing both “conduction efficiency” through myelin dysregulation and “signal sensitivity” via suppression of D1 receptor signaling, thereby converging on the emergence of a social stress-susceptible phenotype.

## 5. Conclusions

This study identified Dnmt3a in NAc as a pivotal regulator of social stress susceptibility, with distinct brain region- and subtype-specific functions. It provided evidences that epigenetic regulation in oligodendrocytes plays a central role in modulating behavioral responses to social stress, and presented a novel framework for understanding the pathophysiology of stress-related psychiatric disorders. Future mechanistic studies could be expanded along three key directions: (1) elucidating the molecular mechanisms by which Dnmt3a regulates MBP and D1 receptors, including direct validation of Dnmt3a binding to the promoter regions of MBP and D1 receptors genes, quantitative assessment of DNA methylation changes at specific CpG sites, and functional confirmation of epigenetically regulated target genes; (2) investigating glia-neuron interactions by selectively manipulating Dnmt3a expression in NAc oligodendrocytes and subsequently monitoring dynamic alterations in neuronal excitability, synaptic transmission efficacy, and circuit-level activity, thereby defining how myelin integrity and neuronal function are coordinately regulated; (3) identifying upstream regulators of Dnmt3a through integrative approaches, including transcriptomic profiling, proteomic interaction screens, and non-coding RNA analyses, to discover critical modulators, such as transcription factors and regulatory RNAs that control Dnmt3a expression in the NAc and integrating these findings into a comprehensive regulatory network. These efforts will deepen the mechanistic understanding of stress susceptibility and accelerate the translation of glial-focused discoveries into novel therapeutic strategies.

## Data Availability

The original contributions presented in this study are included in the article/Supplementary Material. Further inquiries can be directed to the corresponding authors.

## Supplementary Materials

The following supporting information can be downloaded at: https://www.mdpi.com/article/10.3390/biom16050639/s1, Figures S1–S6: The original Western blotting images.

## References

[1] Slavich G.M., Irwin M.R. (2014). From stress to inflammation and major depressive disorder: A social signal transduction theory of depression. Psychol. Bull..

[2] Carnevali L., Montano N., Tobaldini E., Thayer J.F., Sgoifo A. (2020). The contagion of social defeat stress: Insights from rodent studies. Neurosci. Biobehav. Rev..

[3] Yuan M., Yang B., Rothschild G., Mann J.J., Sanford L.D., Tang X., Huang C., Wang C., Zhang W. (2023). Epigenetic regulation in major depression and other stress-related disorders: Molecular mechanisms, clinical relevance and therapeutic potential. Signal Transduct. Target. Ther..

[4] Klengel T., Binder E.B. (2015). Epigenetics of Stress-Related Psychiatric Disorders and Gene × Environment Interactions. Neuron.

[5] Smoller J.W. (2016). The Genetics of Stress-Related Disorders: PTSD, Depression, and Anxiety Disorders. Neuropsychopharmacology.

[6] Sharp T., Collins H. (2024). Mechanisms of SSRI Therapy and Discontinuation. Curr. Top. Behav. Neurosci..

[7] Rafeyan R., Papakostas G.I., Jackson W.C., Trivedi M.H. (2020). Inadequate Response to Treatment in Major Depressive Disorder: Augmentation and Adjunctive Strategies. J. Clin. Psychiatry.

[8] Yang E.J., Rahim M.A., Masieri S., Pasinetti G.M. (2025). Differential susceptibility to repeated social stress induces synaptic plasticity impairment and cognitive deficit in the 5xFAD mouse model. Prog. Neurobiol..

[9] Huang S.H., Liu W.Z., Qin X., Guo C.Y., Xiong Q.C., Wang Y., Hu P., Pan B.X., Zhang W.H. (2022). Association of Increased Amygdala Activity with Stress-Induced Anxiety but not Social Avoidance Behavior in Mice. Neurosci. Bull..

[10] Pessoni A.M., Blanc-Arabe L., Pancotti L., Mansouri S., D’Angelo M., Huot K., Rivera A.M., Peralta M.R., Zhao C., Leboulleux Q. (2025). Transcriptional profiling of the cortico-accumbal pathway reveals sex-specific alterations underlying stress susceptibility. Biol. Psychiatry.

[11] Holt L.M., Gyles T.M., Parise E.M., Minier-Toribio A.M., Rivera M., Markovic T., Yeh S.Y., Nestler E.J. (2025). Astrocytic CREB in Nucleus Accumbens Promotes Susceptibility to Chronic Stress. Biol. Psychiatry.

[12] Gou L., Zheng H., Chen J., Gao Y., Ma J., Chen D., Li Z., Wu C., Lian B., Zhang X. (2025). Social hierarchy and resilience affect stress-induced PTSD via Uba7 gene expression and subsequent inflammation in microglia of the mPFC. Mol. Psychiatry.

[13] Wang Y.T., Li Y., Li M.Y., Zhang Y.M., Wang Y., Xu Q.Q., Liu R.Y., Qin X.Y., Shan Q.H., Wang Y. (2025). Prefrontal corticotropin-releasing factor promotes resilience to social stress. Neuropsychopharmacology.

[14] Montes-Rodríguez C.J., Hernández-Reyes E.D., Piña-Díaz V., Muñoz-Torres Z., Pérez-Zarazúa I., Urteaga-Urías E., Prospéro-García O. (2024). Activity-Dependent Synaptic Plasticity in the Medial Prefrontal Cortex of Male Rats Underlies Resilience-Related Behaviors to Social Adversity. J. Neurosci. Res..

[15] Chen Z., Tang Z., Zou K., Huang Z., Liu L., Yang Y., Wang W. (2021). D-Serine produces antidepressant-like effects in mice through suppression of BDNF signaling pathway and regulation of synaptic adaptations in the nucleus accumbens. Mol. Med..

[16] Xia J., Zou Y., Cui Y., Zhang S., Huo K., Liu W., Huang Z., Zhang Q., Qi Z., Liu W. (2025). Physical exercise activates a PVN-NAc oxytocin circuit to relieve stress-induced depressive-like behaviors. Proc. Natl. Acad. Sci. USA.

[17] Mourtzi N., Sertedaki A., Charmandari E. (2021). Glucocorticoid Signaling and Epigenetic Alterations in Stress-Related Disorders. Int. J. Mol. Sci..

[18] Musazzi L., Mingardi J., Ieraci A., Barbon A., Popoli M. (2023). Stress, microRNAs, and stress-related psychiatric disorders: An overview. Mol. Psychiatry.

[19] Lyko F. (2018). The DNA methyltransferase family: A versatile toolkit for epigenetic regulation. Nat. Rev. Genet..

[20] Rondelet G., Wouters J. (2017). Human DNA (cytosine-5)-methyltransferases: A functional and structural perspective for epigenetic cancer therapy. Biochimie.

[21] Charostad J., Astani A., Goudarzi H., Faghihloo E. (2019). DNA methyltransferases in virus-associated cancers. Rev. Med. Virol..

[22] Duan Z., Lu J. (2020). DNA Methyltransferases in Depression: An Update. Front. Psychiatry.

[23] Demeter K., Török B., Fodor A., Varga J., Ferenczi S., Kovács K.J., Eszik I., Szegedi V., Zelena D. (2016). Possible contribution of epigenetic changes in the development of schizophrenia-like behavior in vasopressin-deficient Brattleboro rats. Behav. Brain Res..

[24] Elliott E., Manashirov S., Zwang R., Gil S., Tsoory M., Shemesh Y., Chen A. (2016). Dnmt3a in the Medial Prefrontal Cortex Regulates Anxiety-Like Behavior in Adult Mice. J. Neurosci..

[25] Morris M.J., Na E.S., Autry A.E., Monteggia L.M. (2016). Impact of DNMT1 and DNMT3a forebrain knockout on depressive- and anxiety like behavior in mice. Neurobiol. Learn. Mem..

[26] Christoffel D.J., Golden S.A., Russo S.J. (2011). Structural and synaptic plasticity in stress-related disorders. Rev. Neurosci..

[27] Huang M., Bao J., Tao X., Niu Y., Li K., Wang J., Gong X., Yang R., Gui Y., Zhou H. (2025). Ventral Hippocampal CA1 GADD45B Regulates Susceptibility to Social Stress by Influencing NMDA Receptor-Mediated Synaptic Plasticity. Neurosci. Bull..

[28] Lu J., Zhang Z., Yin X., Tang Y., Ji R., Chen H., Guang Y., Gong X., He Y., Zhou W. (2022). An entorhinal-visual cortical circuit regulates depression-like behaviors. Mol. Psychiatry.

[29] Li L., Durand-de Cuttoli R., Aubry A.V., Burnett C.J., Cathomas F., Parise L.F., Chan K.L., Morel C., Yuan C., Shimo Y. (2023). Social trauma engages lateral septum circuitry to occlude social reward. Nature.

[30] Kim H.D., Wei J., Call T., Quintus N.T., Summers A.J., Carotenuto S., Johnson R., Ma X., Xu C., Park J.G. (2021). Shisa6 mediates cell-type specific regulation of depression in the nucleus accumbens. Mol. Psychiatry.

[31] Dias C., Feng J., Sun H., Shao N.Y., Mazei-Robison M.S., Damez-Werno D., Scobie K., Bagot R., LaBonté B., Ribeiro E. (2014). β-catenin mediates stress resilience through Dicer1/microRNA regulation. Nature.

[32] Chen Z., Zhang Y. (2020). Role of Mammalian DNA Methyltransferases in Development. Annu. Rev. Biochem..

[33] LaPlant Q., Vialou V., Covington H.E., Dumitriu D., Feng J., Warren B.L., Maze I., Dietz D.M., Watts E.L., Iniguez S.D. (2010). Dnmt3a regulates emotional behavior and spine plasticity in the nucleus accumbens. Nat. Neurosci..

[34] Stadelmann C., Timmler S., Barrantes-Freer A., Simons M. (2019). Myelin in the Central Nervous System: Structure, Function, and Pathology. Physiol. Rev..

[35] Zhang Z., Shu X., Cao Q., Xu L., Wang Z., Li C., Xia S., Shao P., Bao X., Sun L. (2023). Compound from Magnolia officinalis Ameliorates White Matter Injury by Promoting Oligodendrocyte Maturation in Chronic Cerebral Ischemia Models. Neurosci. Bull..

[36] Yamaoka K., Nozaki K., Zhu M., Terai H., Kobayashi K., Ito H., Matsumata M., Takemoto H., Ikeda S., Sotomaru Y. (2025). Neuron-non-neuron electrical coupling networks are involved in chronic stress-induced electrophysiological changes in lateral habenular neurons. J. Physiol..

[37] Bonnefil V., Dietz K., Amatruda M., Wentling M., Aubry A.V., Dupree J.L., Temple G., Park H.J., Burghardt N.S., Casaccia P. (2019). Region-specific myelin differences define behavioral consequences of chronic social defeat stress in mice. eLife.

[38] Maleš P., Pem B., Petrov D., Mangiarotti A., Dimova R., Bakarić D. (2025). Adsorption of Myelin Basic Protein on Model Myelin Membranes Reveals Weakening of van der Waals Interactions in a Lipid Ratio-Dependent Manner. Membranes.

[39] Li Y., Su Z., Zhai J., Liu Q., Wang H., Hao J., Tian X., Gao J., Geng D., Wang L. (2025). Oligodendrocyte-Specific STAT5B Overexpression Ameliorates Myelin Impairment in Experimental Models of Parkinson’s Disease. Cells.

[40] D’Aquila P.S. (2024). Licking microstructure in response to novel rewards, reward devaluation and dopamine antagonists: Possible role of D1 and D2 medium spiny neurons in the nucleus accumbens. Neurosci. Biobehav. Rev..

[41] Fox M.E., Chandra R., Menken M.S., Larkin E.J., Nam H., Engeln M., Francis T.C., Lobo M.K. (2020). Dendritic remodeling of D1 neurons by RhoA/Rho-kinase mediates depression-like behavior. Mol. Psychiatry.

[42] Yin Y.Q., Zhang C., Wang J.X., Hou J., Yang X., Qin J. (2015). Chronic caffeine treatment enhances the resilience to social defeat stress in mice. Food Funct..

[43] Francis T.C., Chandra R., Friend D.M., Finkel E., Dayrit G., Miranda J., Brooks J.M., Iniguez S.D., O’Donnell P., Kravitz A. (2015). Nucleus accumbens medium spiny neuron subtypes mediate depression-related outcomes to social defeat stress. Biol. Psychiatry.

