# Supplementary material for: Cell Type-Specific Downregulation of Dnmt3a in Nucleus Accumbens Oligodendrocytes Prevents Myelin Damage and Reduces Susceptibility to Social Stress in Male Mice
Source: Biomolecules. 2026 Apr 24;16(5):639. doi: 10.3390/biom16050639 (PMC13204232; doi:10.3390/biom16050639)
Supplement: Supplementary file 1 [file biomolecules-16-00639-s001.zip › biomolecules-4210043-supplementary.pdf]

Original Western blot images

Figure S1. The original Western blotting images for Figure 1

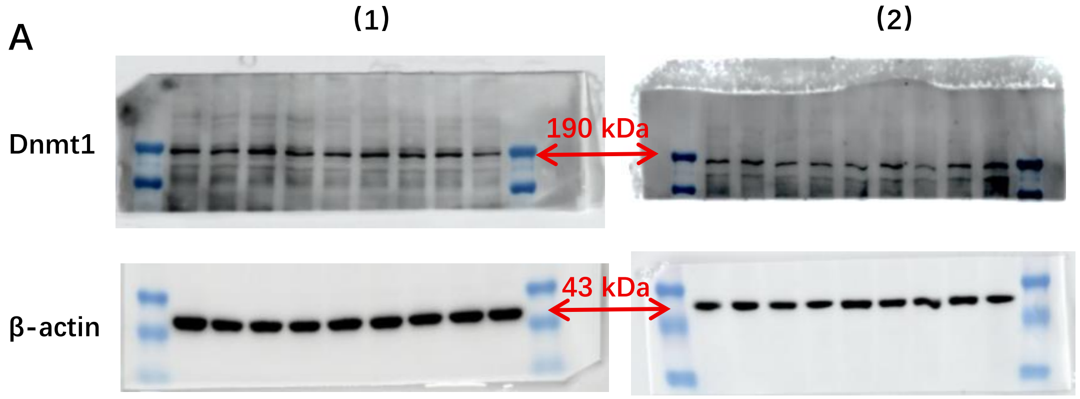

The original Western blotting images for Figure 1D

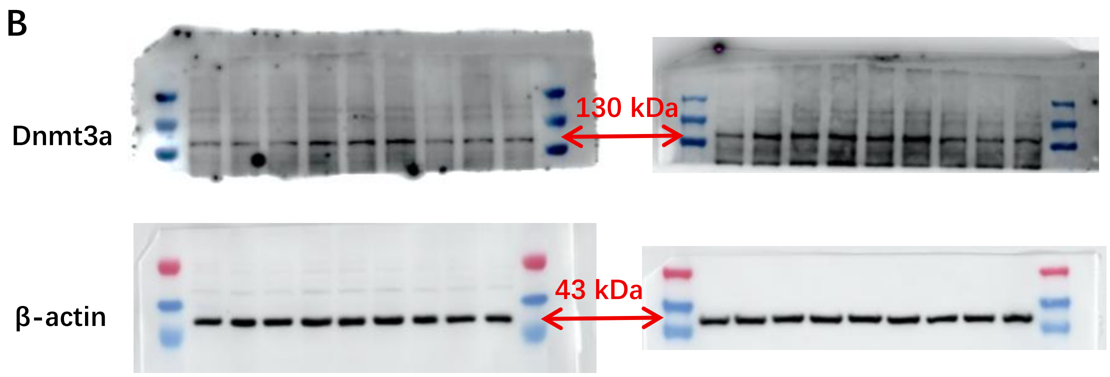

The original Western blotting images for Figure 1E

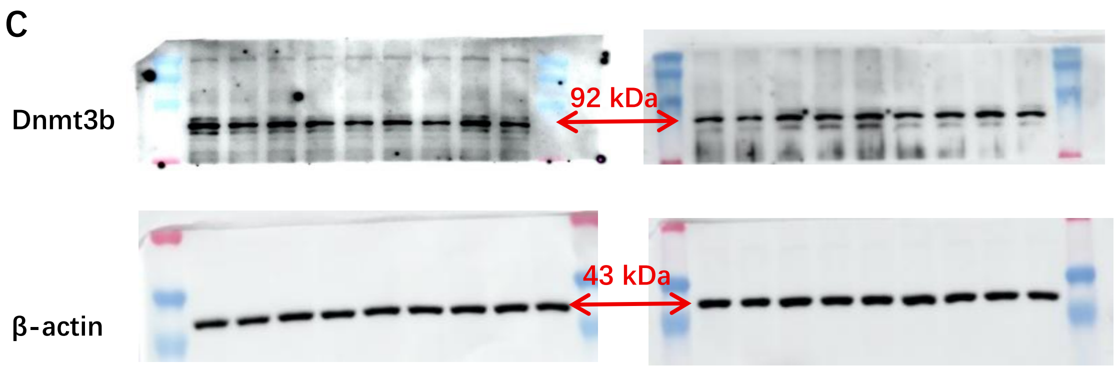

The original Western blotting images for Figure 1F

D

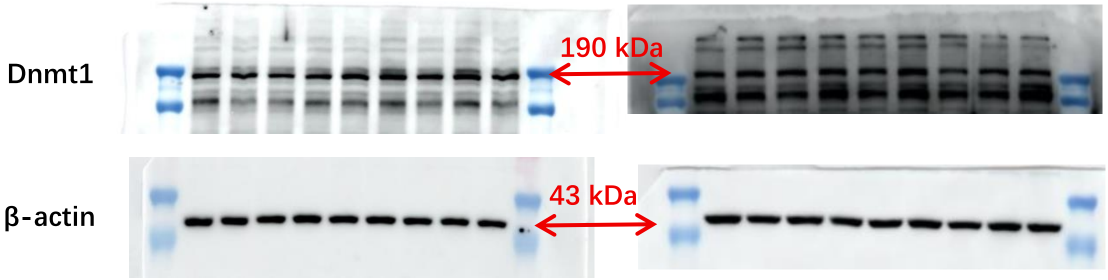

The original Western blotting images for Figure 1G

E

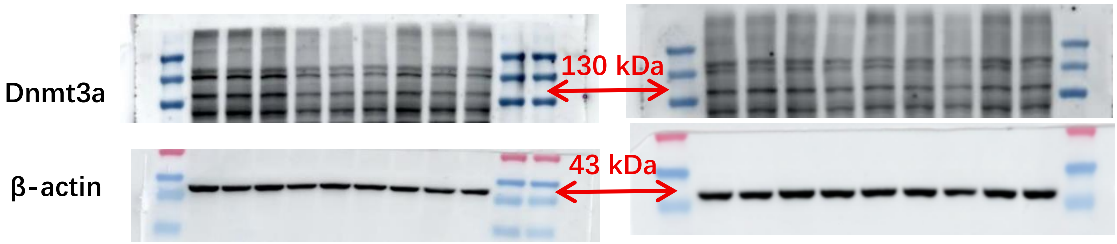

The original Western blotting images for Figure 1H

F

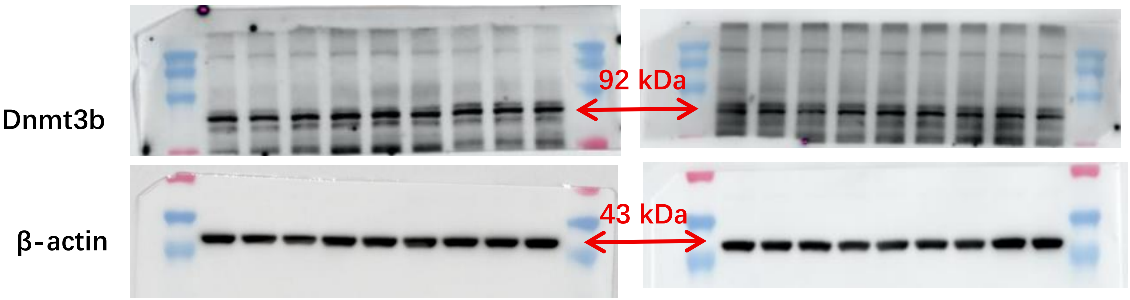

The original Western blotting images for Figure 1I

Figure S2. The original Western blotting images for Figure 2

A

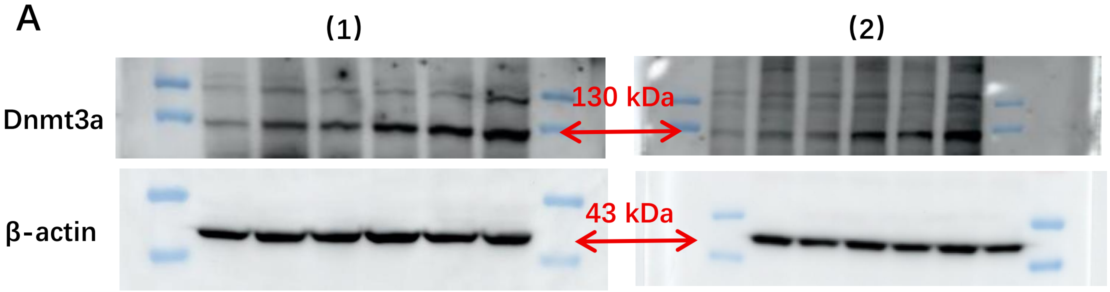

The original Western blotting images for Figure 2C

B

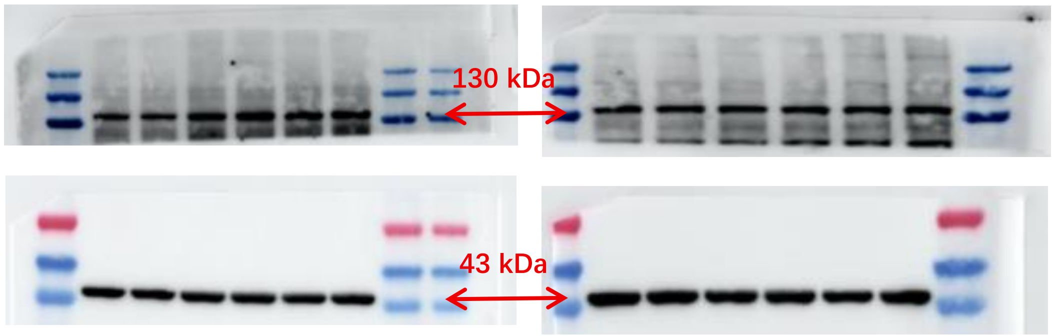

The original Western blotting images for Figure 2R

Figure S3. The original Western blotting images for Figure 3

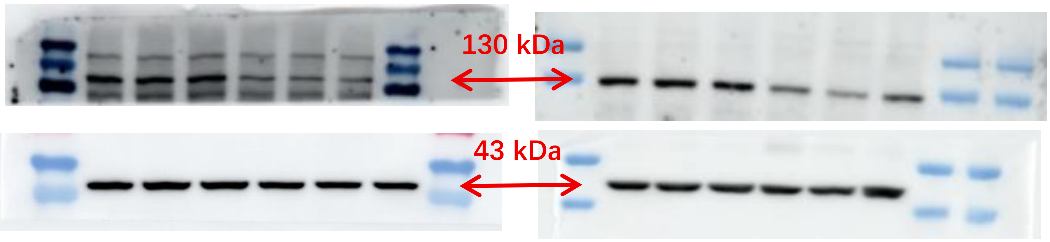

The original Western blotting images for Figure 3C

Figure S4. The original Western blotting images for Figure 5

A

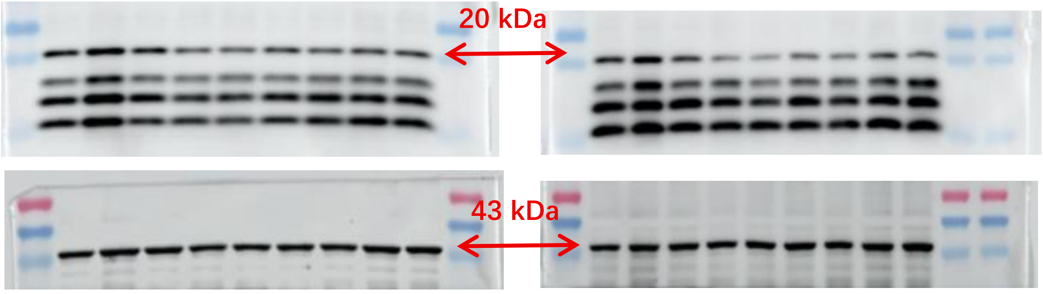

B

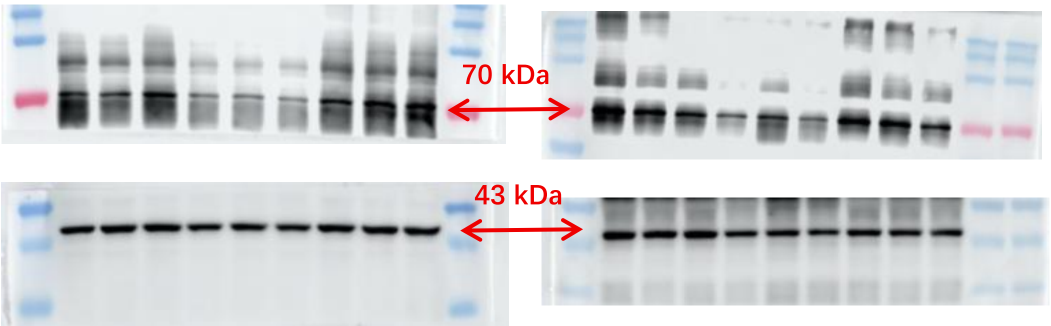

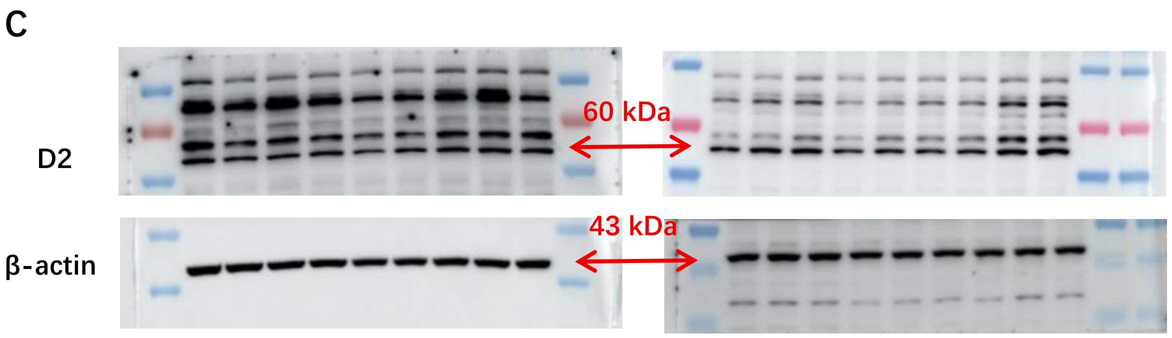

The original Western blotting images for Figure 5F

Figure S5. The original Western blotting images for Figure 6

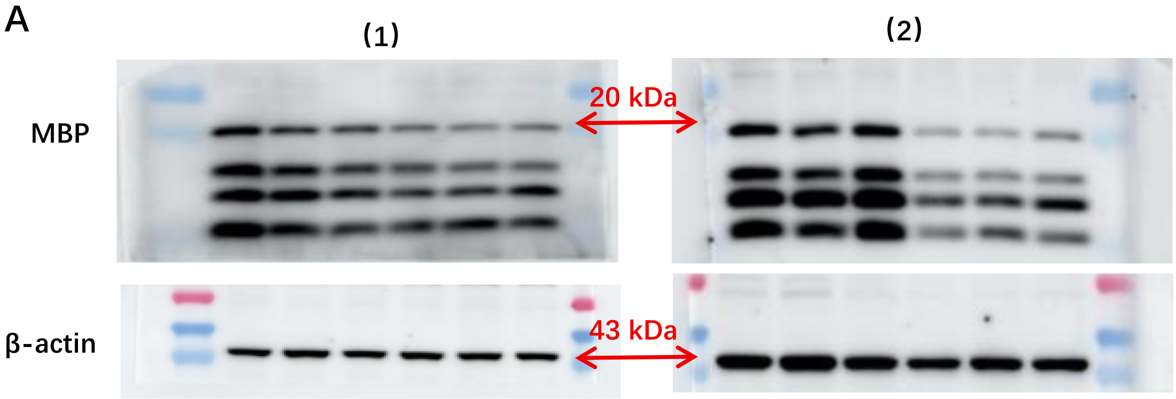

The original Western blotting images for Figure 6A

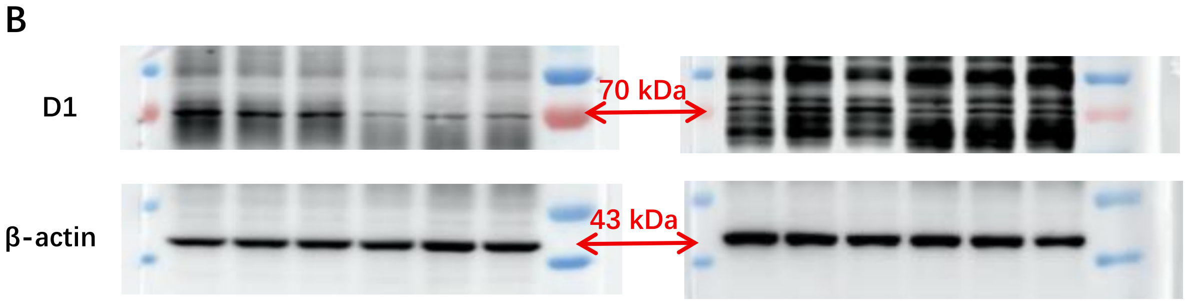

The original Western blotting images for Figure 6B

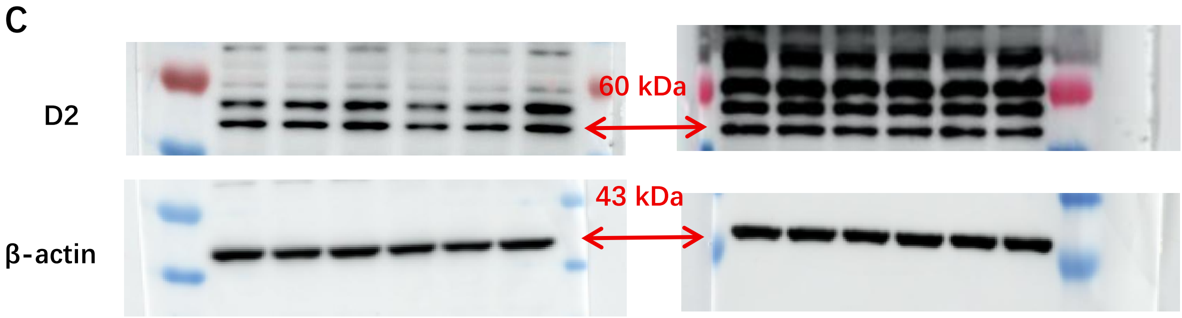

The original Western blotting images for Figure 6C

D

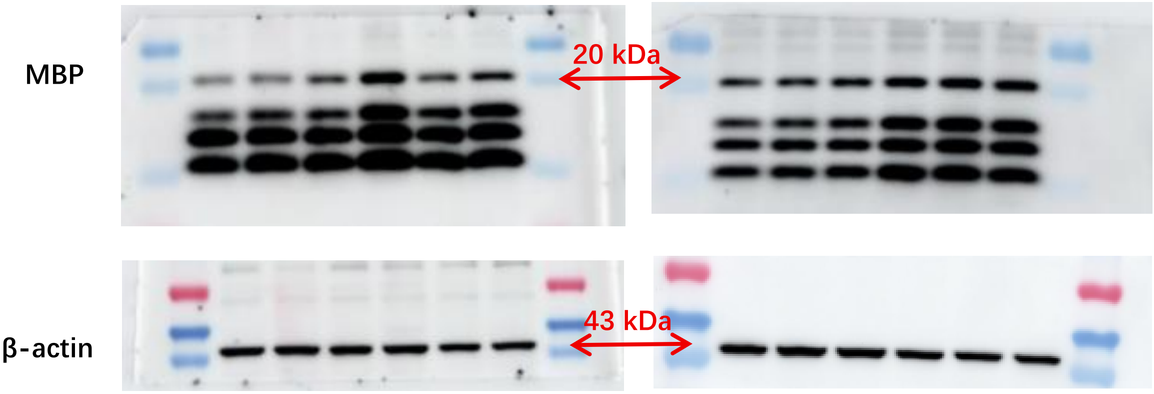

The original Western blotting images for Figure 6D

E

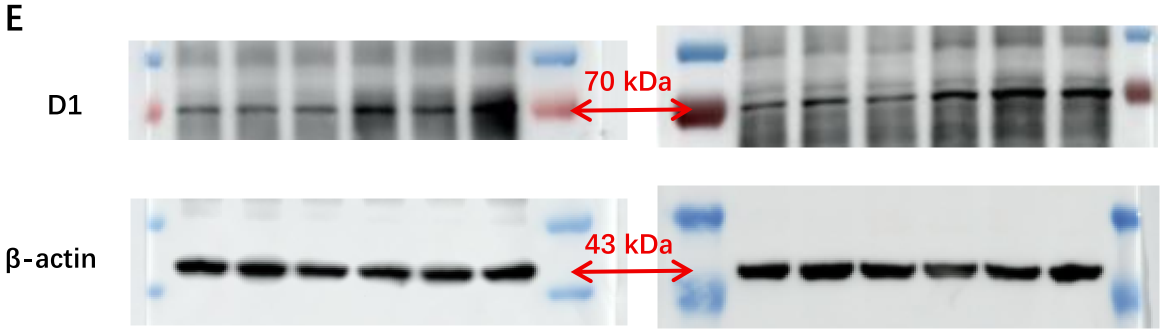

The original Western blotting images for Figure 6E

F

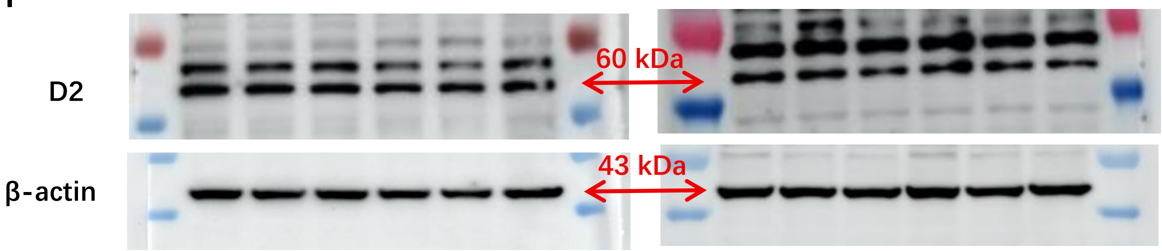

The original Western blotting images for Figure 6F

Figure S6. The original Western blotting images for Figure 7

A

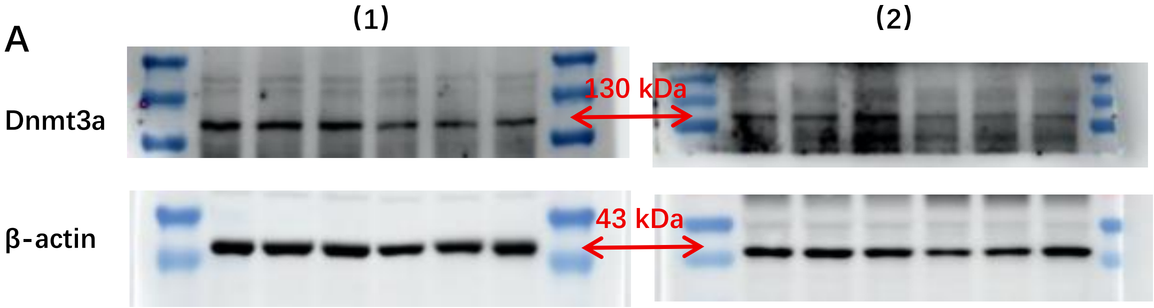

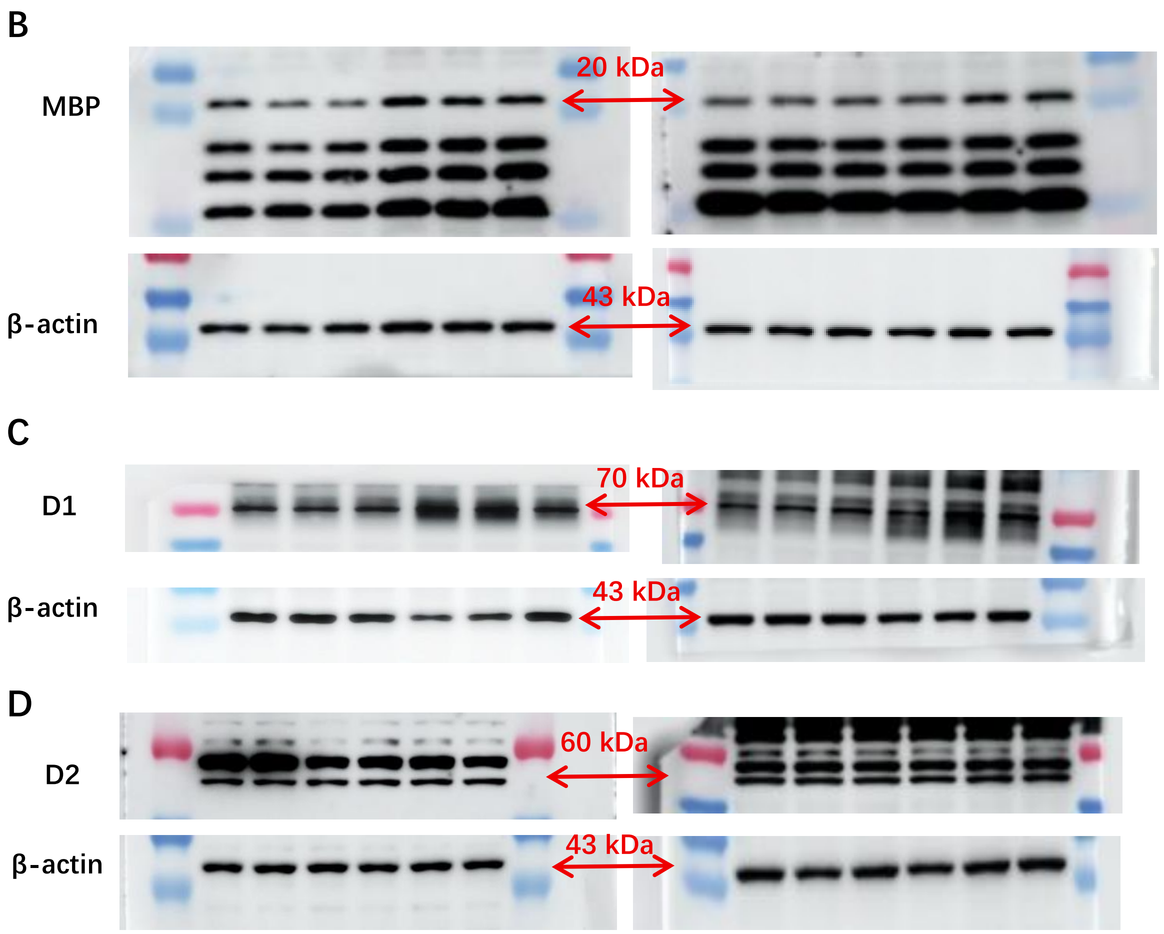

The original Western blotting images for Figure 7O

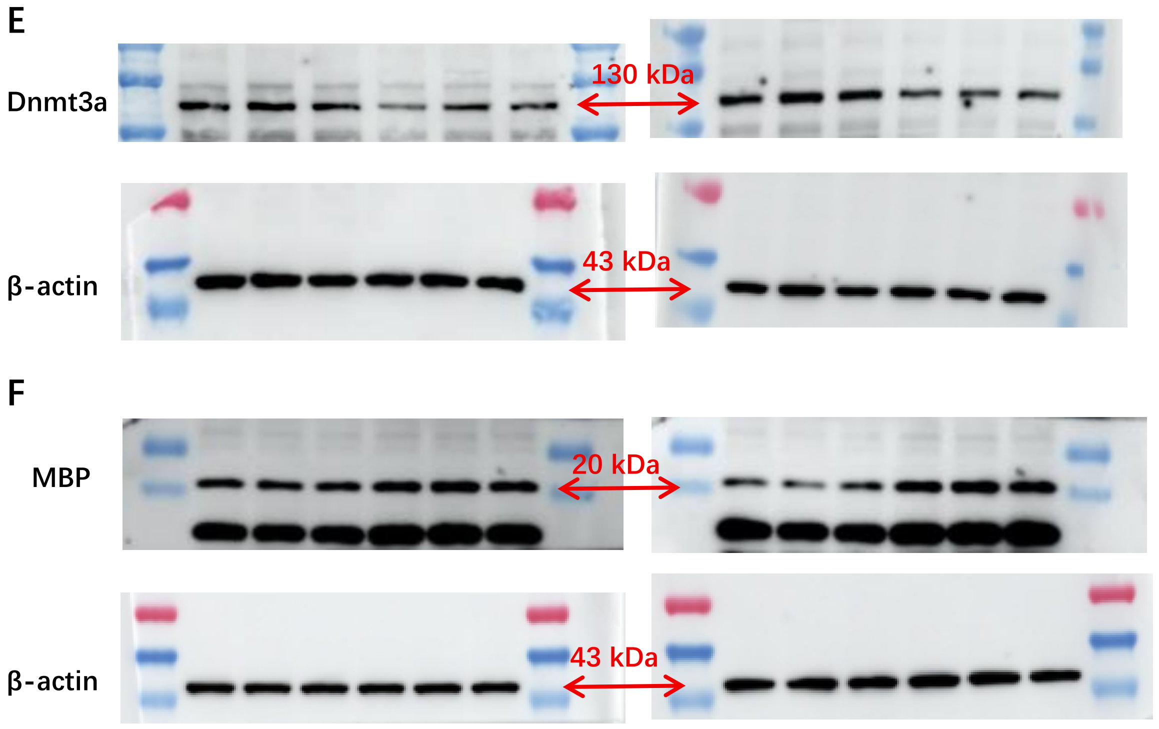

G

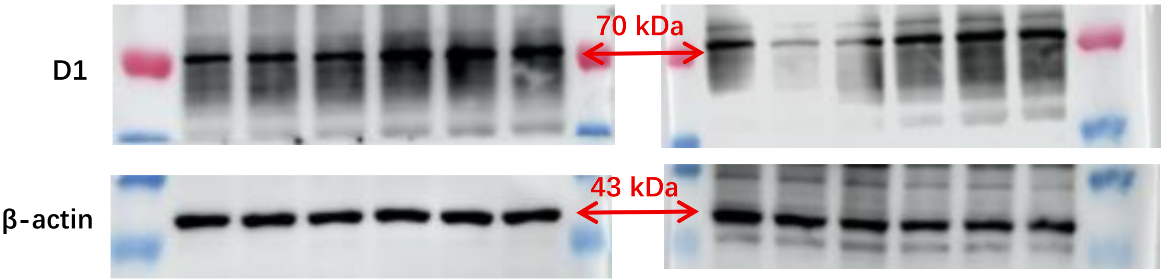

H

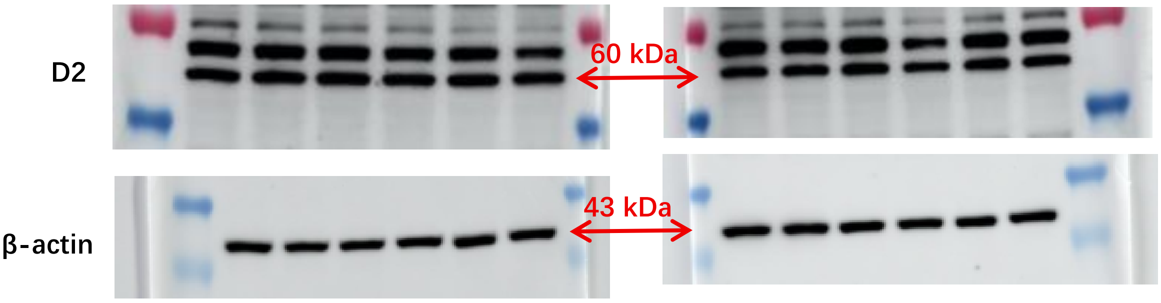

The original Western blotting images for Figure 7T
